# Association between distributed leadership and teachers’ organizational commitment: Evidence from public and private schools in the United Arab Emirates

**DOI:** 10.1371/journal.pone.0351540

**Published:** 2026-06-23

**Authors:** Ali Ibrahim, Shaikha Alshehhi, Nikolaos Tsingilis

**Affiliations:** 1 College of Education, United Arab Emirates University, Al Ain, United Arab Emirates; 2 Ministry of Education, Al Ain, United Arab Emirates; 3 College of Education, United Arab Emirates University, Dubai, United Arab Emirates; University of Science and Technology of Fujairah, YEMEN

## Abstract

Distributed Leadership (DL) has become an important approach in contemporary school reform, promoting teacher agency, shared responsibility, and inclusive decision-making. While DL is linked to school effectiveness, its relationship with teachers’ Organizational Commitment (OC), including Affective (AC), Normative (NC), and Continuance Commitment (CC), remains underexplored, particularly in multicultural contexts such as the United Arab Emirates (UAE). This study’s main purpose was to examine how teachers’ perceptions of DL are associated with their OC in UAE schools. A quantitative, correlational design was employed with a stratified random sample of 1,040 teachers from public and private schools across all seven Emirates. Data were collected using the Distributed Leadership Scale and the TCM Employee Commitment Survey. To support the study objective, measurement analyses were conducted to clarify the structure of the scales. Confirmatory Factor Analysis indicated that a bifactor model provided the best representation of OC across the examined models. Based on consistent empirical results across subsamples and conceptual considerations, two weak CC items were removed, resulting in a more coherent operationalization of the construct. Demographic variables were also examined as contextual factors that may shape the relationship between DL and OC. The findings showed that DL was widely perceived across schools, with public schools reporting slightly higher levels. Structural Equation Modeling indicated a strong positive association between DL and overall OC, particularly for affective and normative commitment, while the association with continuance commitment was weaker. School type showed a limited association with commitment, indicating that DL is more strongly related to teachers’ OC than school type. Demographic variations emerged for nationality, qualifications, experience, school cycle, and school location, but not for gender. The study provides theoretical and practical insights into how DL is associated with teachers’ OC in culturally diverse educational systems and contributes to international literature by supporting the use of these instruments in non-Western settings.

## Introduction

The educational leadership landscape has shifted globally from hierarchical models to more collaborative approaches. In response, Distributed Leadership (DL) has emerged as a promising approach that fosters collective responsibility, collaboration, and capacity building among diverse school stakeholders [1]. Unlike leadership models that centralize authority in a single individual, DL emphasizes the shared responsibility of leadership functions, thereby promoting innovation, trust, and professional growth [2,3].

At the same time, Organizational Commitment (OC) has become a central concern in education due to its relation to teacher motivation, retention, and school effectiveness. Meyer and Allen’s [4] and Meyer, Allen, and Smith’s [5] Three-Component Model (TCM) conceptualizes OC through Affective Commitment (AC)—emotional attachment to the organization, Normative Commitment (NC)—a sense of obligation to remain, and Continuance Commitment (CC)—the perceived cost of leaving. AC and NC are more closely tied to relational and social exchange processes, whereas CC is more strongly associated with perceived costs of leaving [6]. Understanding how leadership practices shape these commitments is crucial, especially in culturally and organizationally diverse educational environments.

The United Arab Emirates (UAE) presents a unique setting to explore these dynamics. Formed in 1971, the UAE comprises seven emirates and operates a dual education system: a public sector serving mostly Emirati citizens under a national curriculum, and a private sector catering primarily to expatriate populations through varied international curricula [7]. The workforce includes educators from diverse nationalities and backgrounds, which creates both opportunities and challenges for school leadership and organizational commitment.

Despite the growing adoption of DL in school improvement efforts, research has not yet sufficiently examined its relationship with teachers’ OC (AC, NC, and CC) in the UAE context. Existing studies have either examined these constructs separately or focused on broader factors such as job satisfaction and school climate [8,9]. Furthermore, the role of teachers’ demographic factors—such as nationality, qualifications, and teaching experience—in shaping these relationships remains insufficiently addressed. Accordingly, this study investigates how teachers’ perceptions of DL relate to their OC across UAE schools and explores the role of school type and demographic variables in shaping this relationship.

### Problem statement and research questions

Despite global recognition of distributed leadership’s positive effects, its relationship with organizational commitment remains underexplored in multicultural educational contexts like the UAE. Existing studies, while valuable, are largely based in relatively homogeneous settings—Belgium [9], Turkey [10], and Tanzania [8]—and do not fully account for the complexities of teacher diversity, curriculum variation, and school governance. Previous research suggests that DL strengthens AC and NC through empowerment and trust [11,12], yet the relationship with CC is inconsistent, often due to measurement limitations or contextual factors [3,6].

In the UAE, Ibrahim and Aljneibi [13] showed that teacher commitment is shaped by personal and workplace factors during reform efforts, but DL and OC have not been examined together. Additionally, the UAE’s education system raises questions about how school type (public and private) and teacher demographics—such as nationality, qualifications, and experience—may shape leadership and commitment dynamics. To date, no study has empirically examined how DL relates to AC, NC, and CC across UAE schools, nor how demographic factors explain these relationships. Addressing this gap, the current study investigates the relationship between teachers’ perceptions of DL and their organizational commitment, while considering the effects of school type and demographic variables.

Therefore, the current study seeks to address the following research questions:

1. What is the underlying structure of teachers’ Organizational Commitment (OC) and perceived Distributed Leadership (DL) in the UAE?
2. To what extent do DL, school type, and teachers’ gender associate with teachers’ OC?
3. To what extent do teachers perceive DL to be practiced by school principals in public and private schools in the UAE?
4. Are there statistically significant differences in teachers’ perceptions of DL practiced by school principals in public and private schools based on demographic characteristics such as teachers’ gender, nationality, years of service, educational qualification, school type, school cycle, and school location?
5. What are the levels of teachers’ OC, including its components of AC, NC, and CC, in UAE public and private schools?
6. Do levels of OC and its subcomponents (AC, NC, and CC) significantly differ according to demographic characteristics such as teachers’ gender, nationality, educational qualification, years of service, school cycle, school type, or school location?

## Literature review and theoretical framework

### Distributed leadership: Concept and empirical insights

Distributed leadership (DL) is defined as a leadership model “stretched over leaders, followers, and their situation” [1,3]. Unlike hierarchical models, DL emphasizes shared authority and situational responsiveness, where leadership responsibilities are distributed across formal and informal actors [14,15]. Harris [16] cautioned that DL was often misinterpreted as task delegation or role expansion rather than genuine redistribution of authority and warned against assuming it to be universally beneficial. She argued that school culture, trust, and context are critical for its success. Later scholarship, however, emphasizes that when enacted authentically—grounded in collaboration, professional trust, and shared decision-making—DL supports cooperation, capacity building, and innovation [2,17,18].

Empirical studies reinforce DL’s value across contexts. In Tanzania, Nguni, Sleegers, and Denessen [8] found that leadership behaviors strengthened teacher satisfaction, while in Turkey DL was associated with greater collaboration and job satisfaction [10,18]. In Chile, Galdames-Calderón [19] highlighted tensions between DL policy and practice but also demonstrated its contribution to collective efficacy and teacher well-being.

Comparative studies add further depth. Kılıçoğlu [20] observed differences between public and private schools in the practice of DL, while Liu et al. [11] highlighted the role of relational trust. Similarly, Uçar [12] confirmed that trust mediates the ways in which DL shapes professional relationships. Collectively, these findings suggest that while DL offers significant benefits, its effects are shaped by governance arrangements, institutional context, and workforce diversity [3].

### Organizational commitment: Dimensions and associations

Organizational commitment (OC) refers to an individual’s psychological attachment to their organization. In education, OC is closely linked to teacher motivation, retention, and effectiveness [21,22]. The widely adopted Three-Component Model (TCM) conceptualizes commitment as comprising three dimensions: Affective Commitment (AC), which reflects an employee’s emotional attachment to the organization; Normative Commitment (NC), which reflects a sense of moral obligation to remain; and Continuance Commitment (CC), which is based on the perceived costs associated with leaving [5,23].

In their meta-analysis of 155 studies, Meyer et al. [6] concluded that AC is the most robust predictor of desirable outcomes, including job satisfaction, performance, and lower turnover intention. CC, by contrast, showed weak or inconsistent associations and in some cases negative implications, while NC was moderately related to outcomes and appeared sensitive to cultural and contextual factors. This distinction highlights that organizational commitment is multidimensional, with each component shaped by different antecedents and associated with different outcomes.

Research has reinforced these distinctions in educational contexts. Mart [24] emphasized the role of passion and dedication in fostering AC, while Klassen and Chiu [25] found that stress, gender, and experience predict differences in teacher commitment and satisfaction. Cross-cultural perspectives add further insight. Hofstede [26] argued that national culture shapes organizational values and responsibilities, with particular relevance for NC in multicultural environments, and in Malaysia, Thien and Adams [27] demonstrated that school leadership is an important predictor of teacher commitment. At the organizational level, Işık [28] found that internal communication strategies enhance teacher commitment, while Guiaselon et al. [29] reported that mismatches between teacher qualifications and subject assignments reduce commitment.

### Linking DL to OC

Scholars have increasingly investigated the specific relationship between distributed leadership and organizational commitment. In Belgium, Devos et al. [9] demonstrated that DL mediates the relationship between principal leadership and teacher commitment, while in Turkey, Akdemir and Ayik [10] confirmed a direct predictive relationship. Similarly, Nguni, Sleegers, and Denessen [8] found that DL was positively associated with teacher job satisfaction and commitment in Tanzania.

Studies also highlight the mechanisms through which DL relates to commitment. Somech [30] showed that participative decision-making both mediates and moderates the effects of leadership on commitment, while Liu, Bellibas, and Printy [11] and Uçar [12] identified relational trust as a central pathway linking DL to professional growth and stronger commitment. Day [21] similarly emphasized that effective leadership fosters teacher commitment by involving them in instructional improvement and collective decision-making.

Beyond leadership mechanisms, demographic and institutional factors may also shape how distributed leadership relates to organizational commitment. Klassen and Chiu [25] showed that individual differences such as stress levels, self-efficacy, and gender predict variation in teachers’ commitment, while Grant [31] similarly observed gendered responses to teacher leadership. At the institutional level, Kılıçoğlu [20] reported that public and private schools demonstrate different patterns of DL practice and its effects on commitment, indicating that governance arrangements and school structures condition the DL–OC relationship. Together, these studies underscore that the relationship of DL with OC is not uniform but contingent on the demographic and institutional contexts in which schools operate.

### Theoretical framework

This study is grounded in Meyer and Allen’s [4] TCM, which conceptualizes OC as comprising AC, NC and CC. AC reflects an emotional attachment to the organization, NC reflects a sense of moral obligation to remain, and CC is based on the perceived costs of leaving [5]. Research has consistently shown that these dimensions respond in different ways to leadership associations: AC has been associated with motivation and instructional quality [22,24], NC with ethical or culturally shaped expectations [6], and CC with job security and contractual conditions.

To examine the leadership relationship with teachers’ OC, this study adopts the lens of DL, conceptualized as a collective, context-responsive process rather than an individual trait [1,15]. The Social Exchange Theory proposed by Blau [32] was used to clarify the psychological mechanisms underpinning organizational commitment. According to this theory, when teachers perceive mutual respect, fair treatment, and professional recognition from their leaders, they develop a stronger sense of loyalty and obligation. In this paper, DL is conceptualized as a relational mechanism that facilitates such reciprocal exchanges. These exchanges are theorized to strengthen AC by fostering emotional connection and professional identification with the school, and NC by reinforcing a sense of obligation rooted in trust and collegiality [11,30]. Accordingly, DL is expected to show stronger associations with affective and normative commitment, which are grounded in relational and social exchange processes, than with continuance commitment, which is more strongly shaped by economic considerations and perceived cost of leaving. This framework provides a basis for understanding how DL operates within the culturally diverse education system of the UAE, where contextual dynamics are likely to shape both distributed leadership practices and teacher commitment.

## Methodology

### Research design

This research used quantitative, correlational, and comparative design. It aimed to examine the relationship between DL and teacher OC, including its three dimensions: AC, NC, and CC. This design was chosen to maintain objectivity, minimize researchers’ bias, and support the examination of latent relationships and group differences through advanced statistical modeling techniques such as Confirmatory Factor Analysis (CFA) and Structural Equation Modeling (SEM) [33,34].

The quantitative approach was well-suited for examining both the measurement structure of the constructs and the relationships between DL and OC across public and private schools in the UAE. By implementing validated instruments, latent variable modeling, representative sampling, and a two-step SEM approach, the design was intended to strengthen the reliability and methodological rigor of the study. These methodological choices enable the study to contribute meaningfully to the international literature related to DL and OC in multicultural educational settings.

### Participants and sampling

The sample consisted of teachers working in both public and private schools across the seven emirates of the UAE. Official statistics released by the Ministry of Education in 2021 [35] estimated that about 21,467 teachers were employed across the country. Using the sample size calculator at Raosoft.com, a minimum of 378 responses was required to achieve a 95% confidence level with a 5% margin of error. However, 1,040 teachers participated in the study, which exceeded the minimum requirement and thereby strengthened the statistical power and breadth of representation within the UAE context.

To obtain a representative sample, a stratified sampling approach with random selection procedures was used to ensure proportional coverage across school types, teaching cycles, and emirates. Although schools were selected using a stratified sampling approach, survey distribution within schools was facilitated through principals and school coordinators to support access to teachers across different emirates and school types. Participation remained voluntary, and teachers were informed that responses would remain anonymous and would not be accessible to school leadership.

The demographic characteristics of the sample reflected the multicultural teacher population of the UAE. Female respondents constituted 79.9% of the sample. Emirati nationals made up 39.1%, Arab expatriates 51.0%, and non-Arab expatriates 9.9%, capturing the diversity of the education sector. Teachers across all educational cycles were represented, including Kindergarten, Cycle 1 (Grades 1–4), Cycle 2 (Grades 5–8), and Cycle 3 (Grades 9–12). Respondents came from both public (72.5%) and private (27.5%) schools.

### Instruments

Data were gathered through two validated instruments. Distributed leadership was measured with Özer and Beycioğlu’s Scale of Distributed Leadership [36]. The scale has ten items rated on a five-point Likert scale (1 = never, 5 = always) that focus on aspects like cooperation, teacher empowerment, and shared decision-making. Prior studies have shown that this measure has high internal consistency (alpha = .92 [37]), which is also the finding of the present dataset (alpha = .92).

Organizational commitment was measured using the TCM Employee Commitment Survey [4], used under license from the University of Western Ontario. Originally developed by Meyer et al. [5] and cited in Meyer and Allen [38], this tool consists of 18 items, six items each for AC, NC, and CC, rated on a seven-point Likert scale (1 = strongly disagree, 7 = strongly agree). A previous study reported reliability coefficients ranging from alpha = .85 to = .89 [18]. In the current study, internal consistency estimates were acceptable for AC and NC, while CC demonstrated comparatively lower reliability, consistent with prior research. Both instruments were administered in English and Arabic to ensure accessibility and cultural appropriateness, following standardized translation and back-translation protocols.

### Data collection procedures

The research was granted ethical approval by the UAE University Social Sciences Research Ethics Committee (Ref: ERSC_2023_4012). The data collection process followed a multi-stage official protocol to guarantee nationwide representativeness. Initially, the Ministry of Education (MoE) sent a formal permission email to all private and public schools across all seven emirates in the UAE, authorizing the survey. To ensure geographical coverage, follow-up permission emails from the MoE were sent directly to school principals. In turn, principals appointed school coordinators to facilitate the distribution of the survey link to all teaching staff. This dual-layered institutional procedure helped ensure that the survey invitation reached teachers across all emirates and facilitated access to a substantial sample of 1,040 participants. Such broad participation would have been difficult to achieve without institutional coordination between 15 February 2024 and 6 May 2024.

The questionnaire included an introductory consent section explaining that participation was voluntary, participants could withdraw at any time without penalty, no identifying information was required, and all responses would remain confidential and used solely for research purposes. To minimize potential response bias associated with school-based distribution procedures, teachers were explicitly informed that participation was voluntary, responses were anonymous, and no identifying information would be shared with school administrators or principals.

All participants were adults (18 years or older). The authors did not have access to information that could identify individual participants during or after data collection. To mitigate the potential for Common Method Bias (CMB) inherent in self-report measures, the measures of distributed leadership and organizational commitment were separated within the instrument to reduce participants’ tendency to provide internally consistent responses across constructs.

### Data analysis strategy

A combination of Confirmatory Factor Analysis (CFA) and Structural Equation Modeling (SEM) procedures was employed to examine the measurement structure of the study constructs and the association between DL and OC. Following the suggestions of Anderson and Gerbing [39], a two-stage approach was adopted. First, the measurement models of the study were examined using CFA to evaluate their underlying structure. Next, structural paths were added to the measurement model. To increase confidence in the results, a cross-validation approach was employed. In particular, the total sample was randomly divided into two equal groups, group A (*n* = 522) and group B (*n* = 521). Preliminary analysis showed that the two groups did not differ in terms of gender (males, females; $\chi^{2}$(1) = 1.57, *p* = 0.211), and school type (public, private; $\chi^{2}$(1) = 1.93, *p* = 0.165). The purpose of this split-sample approach was to examine the stability of item functioning and the consistency of measurement-related decisions across two independent subsamples. Specifically, the study evaluated whether item-retention, item-removal, and theory-consistent model-refinement decisions identified within the calibration sample were supported within the validation sample. This procedure served as an internal robustness check on the stability and consistency of the measurement structure prior to the final structural analyses. Item deletion was retained only when there was convergent psychometric evidence across samples together with conceptual justification. In particular, attention was given to the distinction between high-sacrifice and low-alternative components of continuance commitment, ensuring that retained items reflected a coherent conceptual interpretation of the construct. The removal of weak-performing items was therefore intended to refine the operational representation of continuance commitment rather than redefine the construct itself.

Initially, OC was conceptualized as a multidimensional three-component model [4]. Building on this conceptualization, Meyer et al. [5] developed and validated the widely used TCM Employee Commitment Survey, with 18 items (six per component) and recommended scoring of three correlated subscales. Prior studies have examined the underlying structure of OC and various factorial models have been proposed and tested [40,41]. For example, in their study Cheng and Stockdale [40] compared 12 alternative models such as a unidimensional model, and two- to five-factor models of OC, either oblique or orthogonal. They reported that “Even the one-factor model (model 12) was not appreciably worse than any of the multi-factor, oblique models”. One promising model that received little attention in research on the factorial structure of the OC is the bifactor model [42]. Given that in their study Cheng and Stockdale [40] found that all correlated factor models as well as the one-factor model had similar fit, it seemed reasonable to also examine a bifactor model.

Thus, in the present study three models were postulated and tested; a unidimensional model, a three-correlated factors model, and a bifactor model. The inclusion of a bifactor model was also conceptually motivated, as organizational commitment may reflect both a general underlying attachment to the organization and more specific forms of commitment (affective, normative, and continuance). This approach allows the simultaneous examination of a global commitment factor alongside distinct dimensions, which is particularly relevant in complex organizational settings such as schools. Although the refined measurement structure differs slightly from the original TCM specification, the retained dimensions remained conceptually aligned with Meyer and Allen’s framework, allowing cautious comparison with prior organizational commitment research.

Özer and Beycioğlu [36] suggested that a single latent factor underlies participants’ response to DL. Follow-up studies in various cultural contexts have supported the unidimensionality of DL [43,44]. Thus, in the present study a one-factor model was postulated and tested. Next, a Structural Equation Model was examined with the hypothesis that DL would show a positive association with OC within the structural model. If supported, the bifactor model would suggest that responses to organizational commitment are influenced by both a general factor and specific components, with implications for how the relative importance of AC, NC, and CC is interpreted in subsequent analyses.

Model fit was evaluated using (a) the model chi-square ($\chi^{2}$), (b) the Comparative Fit Index (CFI), (c) the Root Mean Square Error of Approximation (RMSEA), and (d) the Standardized Root Mean Square Residual (SRMR). Following common guidelines, CFI values ≥.95 indicate excellent fit and ≥.90 acceptable fit; RMSEA ≤.06 is considered good and ≤.08 acceptable approximate fit; and SRMR ≤.08 reflects good residual fit [45,46]. Comparisons of nested models were conducted with chi-square difference tests ($\Delta \chi^{2}$) using the corresponding difference in degrees of freedom. In case of non-nested models (e.g., the three-correlated factors model versus the bifactor model), the Akaike Information Criterion (AIC) and Bayesian Information Criterion (BIC) were used. Lower values suggest a better fit.

Independent samples t-tests were used to examine differences in distributed leadership and organizational commitment according to binary demographic variables, including gender and school type. One-way ANOVA was used to examine differences according to nationality, years of experience, qualifications, school cycle, and emirate. Welch’s ANOVA was employed in cases where the assumption of homogeneity of variance was not met, followed by Scheffé post hoc comparisons where appropriate.

The analytical procedure followed a structured sequence designed to align with the study’s research questions and ensure both measurement validity and robust interpretation of findings. First, CFA was conducted to establish the underlying structure of OC and DL and to evaluate the adequacy of the measurement models. Second, SEM was employed to examine the association between distributed leadership, selected contextual variables, and organizational commitment. Third, descriptive statistics were used to examine the overall perceptions of distributed leadership and organizational commitment across UAE schools. Finally, group comparison analyses (independent samples t-tests and one-way ANOVA) were conducted to explore demographic and contextual variations in these constructs.

## Results

### RQ1: Confirmatory factor analysis

Confirmatory Factor Analysis for OC and DL across all examined models are presented in Table 1. With regard to the OC scale, the unidimensional model was far from a good or even a reasonable fit and hence it was rejected. Inspection of the item loadings revealed that the values of #CC10 and #CC11 were not statistically significant and the value of #CC12 was below .30 for group A and #CC11 and #CC12 were not statistically significant, whereas #CC10 yielded a value of .189 for group B. The reason for this might be that conceptually CC as a scale might be made up of two constructs with items 7, 8, and 9 measuring the sacrifice component if a teacher leaves the job, while item 10, 11, and 12 capture aspects of perceived lack of alternatives “too few options to consider leaving this organization” and external constraints “the scarcity of available alternatives” rather than commitment itself. This multidimensionality has been found to attenuate reliability coefficients for the overall CC scale [47].

**Table 1:** Fit statistics for the examined DL and OC underlying structures.

	$\chi^{2}$ (df)	CFI	RMSEA	SRMR	AIC	BIC
DL group A	92.3 (20)	0.945	0.120	0.048	8703.8	8771.9
DL group A mod	74.6 (19)	0.960	0.105	0.043	8660.0	8732.3
DL group B	81.37 (20)	0.945	0.111	0.054	8863.2	8931.3
DL group B mod	70.0 (19)	0.954	0.105	0.051	8843.7	8916.0
DL all	132.1 (19)	0.954	0.109	0.046	17509.2	17593.3
OC Uni group A	1052.6 (135)	0.722	0.131	0.103	33593.4	33746.6
OC 3f group A	829.7 (132)	0.800	0.112	0.109	33242.7	33408.7
OC BiF group A	355.3 (117)	0.933	0.069	0.066	32672.5	32902.3
OC 1Uni group B	923.6 (135)	0.752	0.121	0.092	33104.2	33257.5
OC 3f group B	668.3 (132)	0.838	0.099	0.089	32743.5	32909.5
OC BiF group B	304.5 (117)	0.935	0.055	0.056	32293.4	32523.4
OC BiF all	573.0 (117)	0.935	0.067	0.059	64997.7	65265.0
OC BiF all mod	418.2 (88)	0.949	0.060	0.058	56462.9	56700.5

Note. DL = Distributed Leadership, OC = Organizational Commitment, Uni = Unidimensional Model, 3f = Three Correlated Factor Model, BiF = Bifactor Model

Although the three-correlated factors model showed a better fit in comparison to the unidimensional model ($\Delta \chi^{2}=77.24$, df = 3, *p* < .001 and $\Delta \chi^{2}=110.12$, df = 3, *p* < .001 for the groups A and B respectively), the fit indices did not suggest an acceptable fit. Examination of the item loadings revealed that the values of #CC11 and #CC12 were not statistically significant for the calibration sample and #CC11 for the validation group.

Finally, the bifactor model yielded the best fit indices across the two groups, which were all above or below the widely accepted values. Thus, the bifactor model was selected as the most viable to represent teachers’ response to OC. Omega coefficients were used to calculate subscales’ internal consistency. The results showed acceptable values for the AC and NC subscales (0.856 and 0.835 respectively), but relatively low value for CC (0.603). Inspection of the item-total correlations revealed that items #CC11 and #CC12 have extremely low values (0.055 and 0.196). In light of these findings as well as those from the CFA, it was decided to exclude these items from further analyses. The new omega value for the CC subscale was 0.686, reflecting the “high sacrifice” component of continuance commitment more accurately.

The auxiliary index omega hierarchical for the general (OmegaH) and for the specific factors (OmegaHs) was employed to better understand the bifactor model. OmegaH estimates the proportion of systematic variance in total score that is attributable only to the general factor. Values above .80 suggest that total score behaves as essentially unidimensional, even if the items show multidimensional structure [48]. On the other hand, OmegaHs estimates the proportion of reliable variance in subscale that remains after removing the general factor’s contribution. In the present study, OmegaH = 0.836, and OmegaHs were 0.09, 0.49, 0.17 for the AC, CC and NC subscales respectively. These findings imply that individual differences on the composite are mainly driven by a single common source rather than by the specific factors.

Initial CFA analysis for the DL scale revealed a good fit of the data to the model. However, the RMSEA value was above the suggested cut-off point. Employment of modification indices revealed that the fit of the examined model could be improved if a correlation error was added to the residuals of items 1 and 2. Introduction of this modification and a new run of the analysis showed an improved fit for both the calibration and the validation group. The internal consistency of the eight items was excellent with a value of 0.907. The association of the residuals was statistically significant, yielding a value of 0.28 in the total sample, justifying the inclusion of a correlated error term between item 1 and 2.

### RQ2: Structural equation modeling

Structural Equation Model was fitted to the teachers’ response, in which a regression path from DL to the general factor of OC was introduced. The contribution of two categorical variables of gender (0 = Females and 1 = Males) and school type (0 = Public and 1 = Private) was also examined, by including them in the model as OC predictors. The results showed a good fit, $\chi^{2}$(269) = 874.8, CFI = 0.938, RMSEA = 0.046, SRMR = 0.046. Two of the three exogenous variables were significant explanatory variables associated with OC. In particular, DL showed a strong positive association with OC (β = 1.157, se = 0.115, std β = 0.759, *p* < 0.01). In addition, only the type of school seems to be associated with OC (β = 0.164, se = 0.80, std β = 0.048, *p* = 0.041). Based on the school type, teachers in public schools tend to score higher on OC than those in private schools. Together, the predictors explain 57.0% of the OC variance.

Based on the above results, five composite variables were created representing DL, the OC general factor, and the three OC specific factors (namely AC, CC and NC). It should be noted that the three specific factors were included in subsequent analyses for comparison with prior studies.

### RQ3: Teachers’ perceptions of DL

Descriptive statistics indicated that DL was generally perceived to be practiced at a high level across UAE public and private schools. As shown in Table 2, teachers in public schools reported slightly higher DL perceptions compared with those in private schools. A Welch’s t-test confirmed that this difference was statistically significant, *t*(473.08) = 3.42, *p* = .001, with teachers in public schools reporting significantly higher perceptions of DL.

**Table 2:** Descriptive statistics of DL by school type.

School Type	N	M	SD
Public	755	4.40	0.66
Private	285	4.23	0.72

Note. *N* = 1040; M = mean; SD = standard deviation

### RQ4: Teachers’ Perception of DL across selected demographic variables

A series of independent samples t-tests and one-way ANOVAs were conducted to examine differences in DL perceptions across demographic variables. As summarized in Table 3, DL ratings did not differ by gender. However, significant differences were observed based on nationality, educational qualification, years of service, school cycle, and school location. Emirati teachers reported higher DL perceptions than Arab and non-Arab teachers. Teachers with postgraduate teaching diplomas scored slightly higher than other qualification groups. Teachers with 16–20 years of experience and those in Kindergarten or Cycle 1 reported higher levels of DL perceptions. Additionally, teachers in Ras Al Khaimah and Fujairah perceived more DL compared with those in Sharjah.

**Table 3:** Summary of group differences in DL perceptions.

Variable	Test Type	Test Statistic	p-value	Significant Differences
Gender	t-test	*t*(1038) = 0.02	.983	None
Nationality	ANOVA	*F*(2, 1037) = 9.39	< .001	Emirati > Arab > non-Arab
Educational Qualification	ANOVA	*F*(2, 1037) = 3.09	.046	PG Diploma > Others (slight difference)
Years of Experience	Welch’s ANOVA	*F*(4, 499.89) = 2.74	.028	16–20 yrs > 6–10 yrs
School Cycle	ANOVA	*F*(4, 1035) = 4.77	.001	KG & Cycle 1 > Cycle 3
School Location	Welch’s ANOVA	*F*(6, 299.46) = 4.12	< .001	Ras Al Khaimah, Fujairah > Sharjah

### RQ5: Teachers’ OC between public and private schools

Descriptive statistics indicated generally high levels of OC among teachers in both public and private schools in the UAE. As shown in Table 4, public school teachers reported a slightly higher overall OC than private school teachers though the difference was minor.

**Table 4:** Descriptive statistics of teachers’ OC by school type.

Commitment	Public Schools (*n* = 755)	Private Schools (*n* = 285)
AC	5.76 (1.27)	5.68 (1.21)
CC	4.80 (1.10)	4.66 (1.02)
NC	5.61 (1.20)	5.62 (1.19)
Total OC	5.39 (0.96)	5.32 (0.88)

AC and NC were stronger than CC across both sectors. Teachers in public schools reported higher AC rates than those in private schools while NC levels were virtually identical. CC was the weakest dimension, with teachers in public schools scoring slightly higher than those in private schools. These findings suggest that UAE teachers’ emotional attachment (AC) and moral obligation to stay (NC) were more prominent than staying due to perceived costs of leaving (CC), regardless of school type.

### RQ6: Teachers’ OC across selected demographic variables

Group comparisons revealed that teachers’ OC varied significantly across several demographic characteristics. Gender showed no significant differences in any OC dimensions. However, nationality strongly influenced OC: non-Arab teachers consistently reported lower levels of AC, CC, and NC compared with Emirati and Arab teachers, with the difference between Emirati and Arab teachers not statistically significant.

Educational qualification was also associated with commitment levels. Teachers holding a postgraduate teaching diploma showed the highest OC and subscale scores, while those with Master’s/PhDs reported the lowest levels of OC and CC. Significant differences were also found across teaching experience. Teachers with more than 20 years of experience reported higher AC, NC, and overall OC, while those with 6–10 years of experience showed the lowest scores across most dimensions.

With regard to school cycle, only AC showed statistically significant differences, with Cycle 3 teachers reporting the lowest AC. Teachers from schools with more than one cycle (Mixed cycles) and earlier cycles (e.g., KG, Cycle 1) tended to report slightly higher commitment levels, particularly in AC and NC, though differences in overall OC were not significant.

School location also mattered: Teachers in Ras Al Khaimah reported the highest OC across all subscales, while Sharjah showed the lowest, with a statistically significant difference in OC between these two emirates. Finally, school type (public vs. private) did not produce significant differences in any of the commitment measures.

These results suggest that nationality, education level, experience, school cycle, and location are key demographic factors influencing teachers’ OC in UAE schools (Table 5).

**Table 5:** Summary of group differences in teachers’ OC and its subcomponents across demographic variables.

Variable	Test Type	Test Statistic	p-value	Significant Differences
Gender	t-test	*t*(1038) = 0.94 (AC)	.350 (AC)	None
		*t*(1038) = 1.83 (CC)	.068 (CC)	None
		t(1038)=−0.18 (NC)	.861 (NC)	None
		*t*(1038) = 1.04 (OC)	.298 (OC)	None
Nationality	Welch’s ANOVA	*W*(2, 272.81) = 10.49 (AC)	< .001	Arab > Emirati > non-Arab (AC)
		*F*(2,1037) = 20.47 (CC)	< .001	Emirati/Arab > non-Arab (CC)
		*W*(2,275.84) = 13.15 (NC)	< .001	Arab > Emirati > non-Arab (NC)
		*W*(2,276.28) = 21.70 (OC)	< .001	Arab/Emirati > non-Arab (OC)
Education Qualification	ANOVA	*F*(2,1037) = 3.86 (AC)	.021	PG Diploma > Master’s/PhD (AC)
		*F*(2,1037) = 7.77 (CC)	< .001	Bachelor > Master’s/PhD (CC)
		*F*(2,1037) = 8.06 (NC)	< .001	PG Diploma > Master’s/PhD (NC)
		*F*(2,1037) = 9.45 (OC)	< .001	PG Diploma > Master’s/PhD (OC)
Years of Experience	ANOVA	*F*(4,1035) = 6.88 (AC)	< .001	>20 yrs > 6–10 yrs (AC)
		*F*(4,1035) = 2.05 (CC)	.085	None
		*F*(4,1035) = 3.73 (NC)	.005	16–20 yrs > 6–10 yrs (NC)
		*F*(4,1035) = 4.63 (OC)	.001	>20 yrs > 6–10 yrs (OC)
School Cycle	ANOVA	*F*(4,1035) = 2.70 (AC)	.030	Cycle 2, Mixed > Cycle 3 (AC)
		*F*(4,1035) = 0.26 (CC)	.906	None
		*F*(4,1035) = 2.21 (NC)	.067	None
		*F*(4,1035) = 1.90 (OC)	.109	None
School Location	ANOVA	*F*(6,1033) = 2.94 (AC)	.008	Ras Al Khaimah > Sharjah (OC)
		*F*(6,1033) = 1.96 (CC)	.069	None
		*F*(6,1033) = 2.94 (NC)	.008	Ras Al Khaimah > Sharjah (trend)
		*F*(6,1033) = 3.73 (OC)	.001	Ras Al Khaimah > Sharjah (OC)
School Type	t-test	*t*(1038) = 0.94 (AC)	.350	None
		*t*(1038) = 1.83 (CC)	.068	None
		t(1038)=−0.18 (NC)	.861	None
		*t*(1038) = 1.04 (OC)	.298	None

## Discussion

This study examined DL and teachers’ OC in UAE public and private schools. The findings are discussed in line with the six research questions. The discussion primarily focuses on the structural association between DL, school type, gender, and OC, followed by teachers’ perceptions of DL, differences in these perceptions across demographic factors, levels of OC and its three components, and variations in commitment across demographic variables. This order helps connect the measurement findings with the results and shows how leadership perceptions, commitment patterns, and contextual differences are related within the UAE’s multicultural school system.

### Association between DL and OC

The study confirmed a strong, statistically significant positive relationship between DL and overall teachers’ OC, as shown by a standardized regression coefficient of β

 = 0.759 (p < .01). This indicates that teachers who perceive higher levels of DL from their school leaders are significantly more likely to report stronger commitment to their schools. In addition, the structural model explained 57% of the variance in teachers’ OC, underscoring the substantial association between DL and OC within the present sample. These findings also showed that DL appears to be more strongly associated with teachers’ OC than school type, suggesting that the quality of leadership interactions and shared professional practices within schools may play a more central role in shaping teachers’ commitment than formal institutional classification.

This outcome aligns with Blau’s [32] Social Exchange Theory, which posits that trust, respect, and reciprocal relationships foster employees’ commitment. In school settings, when teachers feel that their contributions are valued through shared decision-making, professional autonomy, and inclusive leadership practices, their sense of emotional attachment and moral obligation toward the school is strengthened. This result is particularly significant within the context of the UAE, where the multicultural composition of the teaching workforce creates a complex environment for leadership practice and professional relationships. In such contexts, DL may function as an important mechanism for fostering inclusion, shared understanding, and professional cohesion across culturally diverse school environments. Shared DL practices may provide a common professional framework that transcends individual cultural differences and reinforces the reciprocal processes emphasized in Social Exchange Theory.

Although the structural model examined the relationship between DL and a general factor of OC, earlier analyses in the current study suggested weaker associations between DL and CC. This weaker association is conceptually expected, as CC often stems from extrinsic considerations such as perceived costs of leaving, job security, or contractual constraints [4], rather than from trust-based or relational processes. The bifactor findings further supported this interpretation, as AC and NC contributed more strongly to the general OC factor than CC. From the perspective of Social Exchange Theory, DL is more strongly associated with socio-emotional bonds and professional reciprocity, which are conceptually closer to AC and NC than to CC. Therefore, the weaker association between DL and CC suggests that DL practices may be more strongly associated with loyalty grounded in appreciation, trust, and professional inclusion than with commitment based on necessity or constraint. However, DL may still indirectly contribute to CC by creating supportive work environments that reduce teachers’ perceived risks associated with leaving, such as opportunities for professional development, collegial support, and inclusion in school decision-making.

These findings support prior studies, such as Leithwood et al. [49], Harris [16,50] and Ibrahim and Aljneibi [13], which demonstrated that DL strengthens teachers’ AC and NC. Similarly, Samancıoğlu et al. [18] reported positive associations between DL and teachers’ OC in Turkish schools. The present study extends this literature by demonstrating that, within the UAE context, DL appears to hold greater importance for teachers’ OC than school type. This suggests that teachers’ lived professional experiences within schools may be shaped more strongly by everyday leadership interactions than by the broader institutional distinction between public and private schools. Nevertheless, the smaller but statistically significant association of school types with OC suggests that institutional conditions, such as school policies, levels of autonomy, or contractual arrangements, may still play a secondary role in shaping teachers’ commitment.

While the statistical magnitude suggests a strong association between DL and OC, it is important to interpret these findings cautiously in light of the study design. As the findings are based on cross-sectional self-report data collected at a single point in time, the observed relationship may partially reflect shared perceptual or method-related variance. Higher perceptions of DL may therefore be associated with stronger perceptions of OC within the same organizational context rather than representing a strictly directional relationship.

Overall, the findings suggest that DL is primarily associated with teachers’ OC through relational and social exchange processes rather than through structural or institutional mechanisms alone. In the UAE context, these findings highlight the potential importance of DL practices in strengthening professional cohesion and OC across culturally diverse educational environments. These findings also reinforce the importance of strengthening DL practices across both public and private schools, particularly within culturally diverse educational settings such as the UAE.

### Teachers’ perceptions of DL across school types

The results found that DL, as perceived by teachers, was widely practiced across public and private schools. Descriptive statistics indicated that teachers in public schools reported significantly higher perceptions of DL (M = 4.40, SD = 0.66) than those in private schools (M = 4.23, SD = 0.72), and this difference was statistically significant, Welch’s t(473.08) = 3.42, p = .001. In addition to reporting higher levels of DL, teachers in public schools also demonstrated less variability in their responses, suggesting more consistent perceptions of DL practices across public school settings.

This finding supports the view that institutional and policy contexts may shape DL practices [1,15]. The more consistent perceptions reported in public schools may reflect the influence of Ministry of Education policies that encourage collaborative decision-making and shared professional responsibilities across government schools. In contrast, the greater variability observed in private schools may be associated with differences in curricula, governance structures, and organizational priorities across the private education sector in the UAE. Kılıçoğlu [20] found similar trends in Turkey, with higher levels of DL reported in public schools. Research has also highlighted the importance of policy and organizational structures in supporting DL practices [19], suggesting that private schools may benefit from clearer leadership frameworks and targeted professional development initiatives to enhance DL practice.

### Demographic associations with teachers’ perceptions of DL

The results revealed that DL is widely recognized within public and private schools in the UAE; nonetheless, significant demographic differences were noted. Gender showed no significant differences in teachers’ DL perceptions, as confirmed by a t-test, t(1038) = 0.02, p = .983, indicating that male and female teachers reported similarly high DL levels. This result corresponds with earlier international research [17,46]. The finding may suggest that DL practices within UAE schools are perceived relatively consistently across male and female teachers, reflecting inclusive professional environments in which leadership participation and collaboration are not strongly differentiated by gender. Similar findings were reported in studies conducted in South Africa [31] and China [36], where DL practices were implemented across diverse school communities.

On the other hand, significant differences were found based on nationality, with Emirati teachers reporting the highest DL perceptions, followed by Arab and then non-Arab teachers, F(2, 1037) = 9.39, p < .001. These patterns may reflect collectivist cultural orientations that place greater emphasis on collaboration, interpersonal relationships, and group belonging [14,26]. In contrast, teachers from more individualist cultural backgrounds may perceive shared leadership practices differently, potentially placing greater emphasis on individual autonomy and role boundaries. Similar patterns related to cultural orientation were found by Thien and Adams [27] in a Malaysian study, emphasizing that cultural values may shape how teachers engage with leadership practices within schools.

Years of experience also shaped perceptions of DL, with teachers in the 16–20 years category reporting significantly higher DL ratings than those with 6–10 years of experience, Welch’s ANOVA F(4, 499.89) = 2.74, p = .028. This is consistent with findings suggesting that more experienced teachers may participate more confidently in leadership processes and collaborative school practices [51,52]. Educational qualifications had a modest yet statistically significant effect, F(2, 1037) = 3.09, p = .046, with teachers holding postgraduate diplomas reporting slightly higher DL perceptions. This suggests that teachers’ perceptions of DL may be associated with academic credentials and opportunities for professional engagement within school leadership processes.

Finally, school cycle and location also revealed significant differences. Teachers in Kindergarten and Cycle 1 reported higher DL perceptions than those in Cycle 3, F(4, 1035) = 4.77, p = .001. Geographic differences were also observed, with teachers in Ras Al Khaimah and Fujairah perceiving significantly more DL than those in Sharjah, Welch’s ANOVA F(6, 299.46) = 4.12, p < .001. These variations likely reflect differences in administrative structures, leadership support, and community engagement across locations.

Overall, these findings suggest that teachers’ perceptions of DL within the UAE are shaped by contextual and demographic characteristics, including cultural background, professional experience, and school setting. The findings further highlight the importance of considering cultural diversity and contextual variation when implementing DL practices in internationalized educational systems such as the UAE.

### Levels of OC among teachers

Teachers reported a generally high level of OC across both public and private schools in the UAE. Public school teachers reported a slightly higher overall OC (M = 5.39, SD = 0.96) than private school teachers (M = 5.32, SD = 0.88), though this difference was relatively minor. Among the three subcomponents, AC and NC scored higher than CC, indicating that emotional attachment and a sense of obligation were more prominent motivators for teachers than remaining due to perceived costs of leaving or lack of alternative opportunities.

Public school teachers reported higher AC (M = 5.76) than private school teachers (M = 5.68), while NC levels were nearly identical (M = 5.61 for public and M = 5.62 for private). CC was the weakest across both groups, with public school teachers scoring M = 4.80 and private school teachers M = 4.66. This pattern suggests that teachers in both public and private schools in the UAE are more strongly motivated by professional attachment, collegial relationships, and internalized loyalty than by perceived costs of leaving or limited employment alternatives. This finding resonates with Ibrahim and Aljneibi’s [13] conclusion that teachers’ OC in the UAE is largely cultivated through professional respect, collegial trust, and supportive leadership practices that strengthen emotional and normative bonds rather than continuance motives.

Internationally, Mart [24] emphasized that teachers’ sense of belonging and professional engagement reinforce OC, while Devos et al. [9] highlighted the role of DL in supporting teacher commitment in Belgium. The present findings similarly suggest that teachers’ OC within the UAE context is more strongly represented by emotional attachment and professional obligation than by continuance-based considerations.

The bifactor findings further supported this interpretation, as the general OC factor was more strongly represented by AC and NC than by CC. This suggests that teachers’ responses in the present study were largely shaped by an overarching sense of attachment to the school, while the three commitment dimensions remained conceptually distinguishable but not equally influential. These findings reinforce the view that OC among teachers in the UAE is primarily grounded in relational and professional experiences within schools rather than in economic or constraint-based considerations alone.

### Demographic associations with teachers’ OC

No gender differences were found across any of the OC subscales—AC, NC, and CC—or overall OC, consistent with Ibrahim and Aljneibi [13] and [53]. This finding may suggest that teachers’ OC is experienced relatively similarly across male and female teachers within UAE schools.

Nationality differences were statistically significant across all OC components and overall commitment, with non-Arab teachers consistently reporting lower levels of AC, NC, and CC, and total OC than their Emirati and Arab counterparts. This aligns with Hofstede’s [26] framework, where collectivist cultures reinforce loyalty, interpersonal relationships, and group obligation. Research suggests that non-Arab teachers’ lower OC may be associated with greater mobility within international job markets, shorter contractual arrangements, and different professional career pathways [53,54]. These factors may contribute to weaker long-term attachment to schools rather than necessarily reflecting deficiencies within school environments themselves. To enhance OC among non-Arab teachers, schools may benefit from strengthening supportive DL practices, professional inclusion, opportunities for career development, and social connectedness within the school community.

Research has shown that leadership practices aligned with DL—such as facilitating teacher autonomy and collaboration—are beneficial for teachers’ OC by enhancing their well-being and perceptions of autonomy [55]. Similarly, fostering social connection and psychological well-being, alongside emotional intelligence, may strengthen teachers’ OC when school leaders enact supportive and facilitative practices within a DL framework [56]. Improving job satisfaction and contractual clarity may also play an important role in supporting continued OC, particularly among teachers working under shorter-term contractual arrangements [57].

Teachers’ qualification differences were also significantly related to all components of OC. Teachers with postgraduate teaching diplomas reported higher OC and subscale scores, especially in comparison to those with Master’s or PhD qualifications. This confirms the findings of Guiaselon et al. [29], suggesting that practical and professionally oriented training may foster stronger OC than more academically or research-oriented qualifications.

Years of experience were also a differentiating factor. Teachers with more than 20 years of experience showed higher AC, NC, and overall OC, while teachers with 6–10 years of experience had the lowest scores across most subscales. These patterns reflect Day’s [21] theory of career stages alongside Meyer et al.’s [6] meta-analysis, both of which suggest that OC may strengthen with professional experience and career maturity.

Differences across school cycle were statistically significant only for AC, where teachers in Cycle 2 and mixed-cycle schools showed higher AC than those in Cycle 3, who may experience weaker emotional attachment because of workload pressures, as suggested by Klassen and Chiu [25]. However, no significant differences were detected for NC, CC, or overall OC across school cycles.

School location showed significant effects on AC, NC, and overall OC. Specifically, teachers in Ras Al Khaimah reported the highest OC levels, while those in Sharjah showed the lowest. This may relate to stronger community relationships, collegial bonds, or leadership practices in smaller emirates, as also reflected in teachers’ responses to the Distributed Leadership Scale in the present study.

No significant differences in any OC dimension or overall OC were found between public and private schools, confirming that school type alone does not appear to explain variation in teachers’ OC. Collectively, these findings suggest that nationality, qualification level, teaching experience, school cycle, and school location are important contextual factors associated with teachers’ OC in the UAE, whereas gender and school type appear less influential.

## Conclusion and limitations

This study’s primary contribution lies in explaining how DL is associated with teachers’ OC in UAE schools. The study examined the relationship between DL of school principals, as perceived by teachers, and teachers’ OC and its components—AC, NC, and CC—across public and private schools in the UAE. The quantitative analyses, supported by validated measurement models, demonstrated that DL showed a strong and significant positive association with OC, with AC and NC contributing most strongly to the overall commitment factor. In contrast, CC showed a comparatively weaker relationship. These findings suggest that teachers’ emotional attachment and moral obligation are more strongly associated with DL practices, whereas CC appears to reflect more structural or contractual considerations. Given the cross-sectional and correlational design of this study, the findings should not be interpreted as causal relationships.

A key methodological contribution of this study lies in the refinement and validation of both measurement scales. The Distributed Leadership Scale was reduced to eight items with strong internal consistency (ω = .91), while problematic items from the CC subscale were removed, improving its reliability (ω = .69). These refinements contribute to ongoing discussions concerning the measurement of DL and OC, particularly within multicultural educational settings.

Distributed leadership was found to be more strongly associated with teachers’ OC than school type, emphasizing that leadership interactions and shared professional practices within schools may play a more central role in shaping teachers’ long-term professional engagement than formal institutional classification alone. Demographic factors such as gender and school type showed limited differences, while nationality, qualification, experience, school cycle, and location yielded meaningful differences. Emirati and Arab teachers demonstrated higher DL perceptions and stronger OC, reflecting the importance of cultural and contextual factors in shaping teachers’ professional experiences within the UAE educational system.

The results of this study underscore the importance of DL as a relational and inclusive framework that may enhance teachers’ emotional and moral commitment. By fostering shared decision-making, recognition, and trust, DL supports teachers as valued contributors to school life. Within the UAE context, DL appears particularly relevant for fostering professional cohesion and strengthening OC across culturally diverse school communities. For policymakers and school leaders, embedding DL principles in leadership development, teacher training, and school improvement strategies may strengthen teacher motivation, reduce turnover, and sustain reform efforts. Strengthening DL practices may also support teachers’ professional inclusion and long-term OC across both public and private schools in the UAE.

Several methodological limitations must be acknowledged. First, the study’s cross-sectional design captured perceptions at a single point in time, which precludes causal inference. Accordingly, the findings should be interpreted as associations rather than causal relationships. Second, the potential for Common Method Bias exists, as both DL and OC were measured through teacher self-reports within a single instrument, although in separate sections. This shared method variance may inflate the observed strength of associations. Future research should consider longitudinal or multi-source designs, such as combining principal-reported leadership practices with teacher-reported OC, to provide a more robust examination of these relationships. In addition, qualitative approaches may offer deeper insight into teachers’ lived experiences of DL and OC within culturally diverse educational settings.

## Supporting information

S1 Data Data Set. (XLSX)
