# Supplementary material for: Association between distributed leadership and teachers’ organizational commitment: Evidence from public and private schools in the United Arab Emirates
Source: PLoS One. 2026 Jun 23;21(6):e0351540. doi: 10.1371/journal.pone.0351540 (PMC13289934; doi:10.1371/journal.pone.0351540)
Supplement: Attachment — Submitted filename: REVIEW-MANUSCRIPT-PONE-D-26-09053.pdf [file pone.0351540.s002.pdf]

REVIEWER COMMENTS

1. Core Contribution and Conceptual Framing

The manuscript takes up a worthwhile and timely topic, but its central contribution is still difficult to locate. As written, the paper seems to be trying to do three things at once: examine the relationship between distributed leadership and organizational commitment, refine the measurement of continuance commitment, and contribute evidence from the UAE context. Any one of these could support a meaningful scholarly contribution. The difficulty is that the manuscript does not yet make clear which of them is doing the main intellectual work, or how they fit together within a single, well-defined argument.

That lack of hierarchy weakens the framing of the study. In some places, the paper reads as a confirmatory test of an established relationship. In others, it moves toward measurement refinement. Elsewhere, it presents itself as a contextually distinctive contribution grounded in the UAE setting. Because these strands are not clearly ordered, the paper feels somewhat split in its purpose, and the reader is left uncertain about what the manuscript most wants to add to the literature.

The paper would become more persuasive if it identified one primary contribution and treated the remaining elements as supporting dimensions of that contribution. If the central aim is to explain the DL–OC relationship, then the scale refinements and the UAE context should be positioned in service of that broader question. If, instead, the real contribution lies in measurement, then the revision of the continuance commitment scale needs to move much closer to the center of the paper. Making that decision would sharpen the introduction, strengthen the interpretation of the findings, and give the manuscript a clearer sense of significance.

2. Theoretical Coherence and Model Justification

The manuscript brings together distributed leadership, the Three-Component Model of organizational commitment, and Social Exchange Theory, but these elements are not yet woven into a framework that fully explains the structural model being tested. The ingredients are present, yet the theoretical logic that connects them remains underdeveloped.

The discussion of the links between distributed leadership and the three dimensions of commitment stays fairly general. The manuscript suggests that these relationships exist, but it does not sufficiently explain the mechanisms through which they are expected to emerge. That leaves the model looking more empirically assembled than theoretically generated.

This is especially noticeable in the treatment of affective, normative, and continuance commitment. The manuscript states that distributed leadership should relate differently to each dimension, but those differences are not really derived from theory. Social Exchange Theory is invoked, though its role remains mostly implicit, and the reader is not shown clearly how it informs the specific paths estimated in the SEM.

The theoretical framing would be much stronger if these links were made more explicit. The manuscript could, for instance, explain how distributed leadership fosters relational conditions such as trust, reciprocity, and shared responsibility, which in turn are more likely to nourish affective and normative commitment than continuance commitment. A more direct alignment between theory and

model specification would deepen the explanatory force of the paper and make the structural analysis feel more fully justified.

3. Analytical Design and Internal Consistency

The manuscript moves through several analytical stages—descriptive comparison, measurement refinement, and structural modeling—but the relationship among these stages is not yet fully clear. In particular, the paper relies on the full scales for descriptive analyses and then shifts to modified scales, after item removal and model adjustment, for the SEM. That transition is important, but it is not explained in enough depth.

As a result, the study develops an internal inconsistency in the way its key constructs are operationalized. It becomes difficult to tell whether the structural findings should be read as evidence about the original constructs or about revised versions of those constructs. That ambiguity matters because it affects how the results can be compared across research questions and across different parts of the paper.

The manuscript would benefit from a clearer explanation of the analytical logic that connects these phases. The authors should spell out why different versions of the measures were used at different stages, whether the SEM results depend on a reconceptualization of the constructs, and how readers should understand the relationship between the descriptive and structural findings. A more explicit account of that progression would make the analysis feel more coherent and easier to interpret.

4. Measurement Integrity and Model Modifications

The measurement revisions are central to the manuscript's argument, yet they are not fully justified or interpreted. Removing CC11 and CC12, and introducing correlated residuals within the distributed leadership scale, may improve model fit, but at present those decisions appear to be driven mainly by statistical convenience rather than conceptual reasoning.

That creates a problem for construct validity. The manuscript does not adequately address whether dropping the continuance commitment items changes what the construct now represents, nor does it explain how the revised version compares with the original validated scale. The same issue arises with the correlated residuals: they are introduced as a way of improving fit, but the paper does not offer a substantive explanation of what those shared residual relationships mean. Without that explanation, it is difficult to know whether the changes reflect theoretically meaningful overlap or simply model-specific adjustment.

The bifactor model also deserves fuller interpretation. The results seem to indicate a strong general factor, yet the manuscript continues to discuss the three commitment dimensions as though their distinctiveness were unproblematic. That tension is important and should be addressed directly.

This section would be stronger if the manuscript offered a conceptually grounded rationale for each measurement decision and then discussed the implications of those decisions more openly. The paper needs to clarify whether the revised scales still represent the same constructs, how they should be interpreted in relation to prior studies, and whether the bifactor results ultimately support a multidimensional or more largely unidimensional understanding of organizational commitment.

5. Inferential Validity and Interpretation of Results

The interpretation of the findings sometimes goes beyond what the design can reasonably support. The manuscript repeatedly uses terms such as “predicts” and “influence,” which suggest directional or causal inference, even though the data are cross-sectional and based on perceptions collected at a single point in time. SEM does not, by itself, remove that limitation, and the current wording risks overstating the evidentiary strength of the study.

The reported relationship between distributed leadership and organizational commitment is also notably strong, yet the manuscript does not examine that strength with sufficient caution. Because both constructs are measured through self-report in the same instrument, some of the observed association may reflect shared method variance or perceptual alignment rather than a purely substantive relationship.

The discussion would be more convincing if the manuscript adopted more careful, associational language and engaged more directly with the magnitude of the observed relationships. This means acknowledging the possibility of common method bias, considering whether the strength of the association may be somewhat inflated, and being more explicit about what the design allows the authors to claim and what it does not.

6. Empirical Interpretation and Substantive Insight

The results are reported with statistical detail, but the manuscript does not yet extract enough substantive meaning from them. The paper shows a strong association between distributed leadership and organizational commitment and a much weaker role for school type, yet these patterns are largely described rather than interpreted.

What is missing is a deeper explanation of why leadership perceptions seem to matter so much more than structural characteristics such as school type. That pattern could reflect something important about the lived organizational environment of schools, the salience of leadership in shaping professional experience, or the nature of the measures themselves. At present, however, the manuscript does not pause long enough to consider those possibilities.

A similar issue appears in the treatment of continuance commitment. Its weaker performance is acknowledged, but not really unpacked, even though the paper itself recognizes earlier that continuance commitment is often conceptually unstable. That leaves a potentially interesting finding underdeveloped.

The manuscript would gain depth by moving beyond statistical reporting toward fuller substantive interpretation. The discussion should explain why some relationships are especially strong, why others are limited, what these patterns imply for leadership and commitment in practice, and how they speak back to the literature rather than simply sit alongside it.

7. Contextual Contribution (UAE Setting)

The UAE is presented as an important and distinctive setting, but the manuscript does not yet make full analytical use of that context. At the moment, the setting is described more than it is interpreted.

In its current form, the paper shows that the DL–OC relationship is present in the UAE, but it does not go far enough in explaining whether the UAE context changes the meaning, strength, or

implications of that relationship. Given the multicultural composition of the teaching workforce and the coexistence of public and private school systems, the context has the potential to generate genuinely useful insight. That potential, however, is only partially realized.

The contextual contribution would become much stronger if the discussion connected the findings more directly to features of the UAE educational landscape. For example, the manuscript could consider how workforce diversity, institutional arrangements, governance structures, or school sector differences shape leadership practice and commitment patterns, and whether the findings confirm or complicate what has been reported in other settings.

8. Methodological Transparency and Reporting

8.1 Sampling Strategy and Representativeness

The manuscript describes the sampling design as a combination of random and stratified sampling, but the actual recruitment process appears more mediated than that description suggests, since survey distribution was conducted through principals and school coordinators. The relationship between the intended design and the implemented procedure therefore needs clearer explanation.

A more transparent account of how participants were reached, how evenly the sample was distributed across the stated strata, and whether any selection effects may have emerged would strengthen confidence in the representativeness of the sample.

8.2 Data Collection Context and Response Conditions

The ethical procedures are clearly stated, but the manuscript says less about the practical context in which the survey responses were collected. Because the surveys were distributed through school leadership, some participants may have felt an implicit expectation to take part, or may have shaped their responses with that context in mind.

It would help to clarify more explicitly how anonymity and voluntariness were protected in practice, and whether any steps were taken to reduce the possibility of response bias in a school-based administrative setting.

8.3 Measurement Approach and Self-Report Dependence

All of the key variables—distributed leadership and organizational commitment—are measured through self-report at a single point in time. That design choice raises the possibility of common method bias and perceptual alignment, especially given the strength of the relationships reported.

The manuscript would be more methodologically balanced if it acknowledged this limitation directly and reflected on how the use of perception-based measures shapes the interpretation of the findings.

8.4 Analytical Procedures and Assumptions

The manuscript justifies the use of parametric tests on the basis of sample size, but the reporting of assumptions such as normality, homogeneity, and outlier handling remains relatively thin. Some procedures are mentioned, yet not in enough detail for the reader to fully judge analytical robustness.

Providing clearer information about assumption testing, the criteria used for outlier removal, and the handling of any departures from assumptions would improve transparency and reproducibility.

8.5 Model Specification and Validation Strategy

The combined use of CFA, SEM, and split-sample validation is one of the study's stronger methodological features. Even so, the manuscript would benefit from clearer reporting of how decisions were made across the calibration and validation samples, especially in relation to item removal and model modification.

The paper should clarify whether modifications were identified in the calibration sample first and then tested in the validation sample, since that sequence matters for judging the stability of the model.

8.6 Integration of Analytical Stages

The manuscript uses a range of analytical techniques, including descriptive statistics, ANOVA, regression, and SEM, but the transitions between these stages are not always well explained. The move from more descriptive or exploratory analysis to confirmatory modeling would be easier to follow if the paper made clearer how each analytical stage prepares the ground for the next.

A stronger narrative thread across these stages would improve the overall methodological coherence of the manuscript.

9. Overall Integration

The manuscript has several genuine strengths in terms of dataset quality, topical relevance, and analytical ambition. At present, however, those strengths do not fully come together because the major components of the study are not yet aligned as tightly as they could be. The theoretical framing, measurement decisions, analytical strategy, and interpretation of findings sometimes seem to move alongside one another rather than reinforcing a single, unified argument.

This is why the paper can feel fragmented even though it contains many of the elements of a strong study. It is not that the manuscript lacks substance; rather, the substance is not yet fully coordinated.

The paper would be significantly stronger if it tightened the alignment across the full research process. That means ensuring that the theoretical framework genuinely supports the tested model, clarifying how measurement decisions reshape the constructs under analysis, organizing the analytical stages so that each one builds logically on the previous one, and calibrating the interpretation of the findings to the limits of the design and data.

With that level of integration, the manuscript could move from being a technically capable study with several promising parts to a more coherent and compelling scholarly contribution.

Final Recommendation

Major Revision

The manuscript is built on a substantial empirical dataset, uses appropriate statistical techniques—including CFA and SEM with cross-validation—and addresses a topic that is clearly relevant within educational leadership research. Those features give the study a strong foundation and suggest that it has real potential to make a useful contribution to the literature. In its current form, however, the manuscript has not yet reached the level of conceptual clarity, analytical coherence, and interpretive precision needed for publication.

The central issue is not a lack of effort, nor a weakness in the dataset itself. The more pressing concern is that the manuscript is operating across several analytical agendas at once—structural modeling, measurement refinement, and contextual contribution—without yet organizing them into a single, clearly articulated scholarly purpose. As a result, the theoretical framing, measurement choices, analytical procedures, and interpretation of results do not consistently pull in the same direction.

Several areas therefore need careful revision. The theoretical framework does not yet do enough to generate and justify the structural model. The measurement modifications are introduced without fully addressing what they mean for construct validity. And the interpretation of the findings sometimes reaches beyond what a cross-sectional, perception-based design can sustain. None of these issues makes the study unpublishable in principle, but together they limit its present credibility and explanatory force.

The encouraging part is that these are remediable problems. The manuscript would improve substantially if it defined a clear primary contribution and aligned the paper around it, strengthened the connection between theory and model specification, offered more transparent and conceptually grounded justification for its measurement decisions, and recalibrated its claims so they remain proportionate to the design. With that level of revision, the study could become not only technically sound but also intellectually persuasive. At this stage, though, major revision is the appropriate recommendation.
