# Supplementary material for: Association between distributed leadership and teachers’ organizational commitment: Evidence from public and private schools in the United Arab Emirates
Source: PLoS One. 2026 Jun 23;21(6):e0351540. doi: 10.1371/journal.pone.0351540 (PMC13289934; doi:10.1371/journal.pone.0351540)
Supplement: Attachment — Submitted filename: Response to Reviewers.docx [file pone.0351540.s004.docx]

**Responses to the editor’s comments**

We sincerely thank the editorial office for the careful review of our submission and for the opportunity to revise the manuscript. We carefully addressed all editorial requirements and revised the submission accordingly. Detailed responses are provided below.

**1. Response to Reviewers Letter**

**Response:**

A detailed point-by-point “Response to Reviewers” document has been prepared and uploaded under the appropriate file designation (“Response to Reviewers”). The document includes responses to all comments raised by the reviewers, together with descriptions of the revisions made throughout the manuscript.

**2. Manuscript File Format and Submission Files**

**Response:**

The manuscript was converted to LaTeX using the official PLOS ONE LaTeX template. In accordance with PLOS ONE submission requirements, the compiled manuscript PDF has been uploaded as the main manuscript file, and the corresponding LaTeX source (.tex) file has been uploaded under the file designation “Other.” In addition I also uploaded a clean Word file and another Word file with changes highlighted in yellow.

**3. PLOS ONE LaTeX Template Compliance**

**Response:**

The manuscript was carefully reformatted using the official PLOS ONE LaTeX template. The revised submission was reviewed to ensure consistency with PLOS ONE formatting requirements, including manuscript structure, references, tables, figures, and supporting information formatting.

**4. Data Availability Statement and Minimal Dataset**

**Response:**

A fully anonymized and de-identified dataset containing the data underlying the reported analyses has been included as Supporting Information in accordance with PLOS ONE data-sharing requirements. All potentially identifying information, metadata fields, and unnecessary identifiers were removed prior to resubmission. The Data Availability Statement was also revised to clarify that all relevant data underlying the findings are provided within the manuscript and Supporting Information files.

**5. Authorship and Affiliations**

**Response:**

The authorship list and institutional affiliations were carefully reviewed and updated in both the manuscript and the Editorial Manager submission system. All authors are now correctly linked to their institutional affiliations, and the corresponding author has been clearly identified in accordance with PLOS ONE author guidelines.

==================================================================================

**Response to Reviewers**

We sincerely thank the reviewers for their careful evaluation of our manuscript entitled “Distributed Leadership as a Predictor of Teachers' Organizational Commitment: Evidence from Public and Private Schools in the United Arab Emirates” now retitled “Association between distributed leadership and teachers' organizational commitment: Evidence from public and private schools in the United Arab Emirates.” We are grateful for the thoughtful and constructive feedback, which significantly strengthened the manuscript conceptually, theoretically, methodologically, and analytically.

In response to the comments, we undertook a substantial revision of the manuscript. The revised version now presents a clearer and more coherent central contribution focused on the association between distributed leadership (DL) and teachers’ organizational commitment (OC), while positioning the measurement refinement procedures and the UAE contextual contribution as supporting dimensions of that broader aim. We also strengthened the theoretical integration between distributed leadership, the Three-Component Model of organizational commitment, and Social Exchange Theory; clarified the analytical logic connecting CFA, SEM, descriptive analyses, and group comparisons; expanded the conceptual justification for measurement decisions; recalibrated causal language to associational language; and deepened the substantive interpretation of findings within the multicultural educational context of the UAE.

Below, we provide a detailed point-by-point response to each comment.

**REVIEWER #1**

1. **Core Contribution and Conceptual Framing**

**Comment:**
The manuscript appeared to pursue multiple overlapping purposes simultaneously, including examining the DL–OC relationship, refining measurement, and contributing UAE evidence, without clearly identifying the primary contribution.

**Response:**
We appreciate this important observation and substantially revised the manuscript to establish a clearer hierarchy among the study’s contributions.

The revised manuscript now explicitly positions the association between distributed leadership and teachers’ organizational commitment as the primary contribution of the study. The measurement refinement procedures and the UAE contextual contribution are now framed as supporting dimensions that strengthen the interpretation of this central relationship rather than functioning as independent research agendas.

To address this concern, we revised the:

- Abstract,
- Introduction,
- Problem Statement,
- Research Questions,
- Data Analysis Strategy,
- Discussion,
- and Conclusion.

The Introduction now explicitly frames the study around the DL–OC relationship, while the Discussion and Conclusion sections were reorganized to ensure that all findings are interpreted in relation to this central argument.

For example, the revised conclusion now opens with:
“This study’s primary contribution lies in explaining how distributed leadership is related to teachers’ organizational commitment in UAE schools.”

These revisions were intended to improve conceptual coherence and align the theoretical framing, analytical procedures, and interpretation around a single unified scholarly contribution.

2. **Theoretical Coherence and Model Justification**

**Comment:**
The theoretical framework did not sufficiently integrate distributed leadership, the Three-Component Model, and Social Exchange Theory into a coherent explanatory framework.

**Response:**
We thank the reviewer for this important observation and substantially revised the theoretical framework and discussion sections to strengthen theoretical integration.

Specifically:

- the manuscript now explains more explicitly how distributed leadership may foster relational conditions such as trust, reciprocity, shared responsibility, collegiality, inclusion, and professional recognition;
- these mechanisms are now directly linked to affective commitment and normative commitment through Social Exchange Theory;
- and the weaker relationship with continuance commitment is now interpreted as conceptually expected because continuance commitment is more strongly associated with perceived costs of leaving and structural considerations rather than relational exchange processes.

The revised manuscript now explicitly explains:
“Distributed leadership is conceptualized as a relational mechanism that facilitates reciprocal exchanges characterized by trust, recognition, and shared professional responsibility.”

The SEM discussion was also revised to explain why distributed leadership appears more strongly associated with affective and normative commitment than with continuance commitment. These revisions were intended to ensure that the structural model appears theoretically generated rather than empirically assembled.

3. **Analytical Design and Internal Consistency**

**Comment:**
“The manuscript moves through several analytical stages…would make the analysis feel more coherent and easier to interpret.”

**Response:**
We thank the reviewer for pointing out this issue. We agree that the previous version of the manuscript was not sufficiently clear. In response, we restructured both the Data Analysis Strategy and Results sections to improve the coherence and interpretability of the analytical sequence.

Specifically:

- the revised manuscript now begins with Confirmatory Factor Analysis (CFA) to examine the underlying structure and psychometric adequacy of the variables under study;
- this is followed by Structural Equation Modeling (SEM) examining the associations between distributed leadership and organizational commitment;
- and finally descriptive and group comparison analyses are presented to explore demographic and contextual variation.

We also clarified the relationship between the descriptive analyses and the latent structural analyses. The revised manuscript now explicitly explains that:

- CFA was used to establish the measurement structure and evaluate the adequacy of the scales;
- SEM relied on the refined latent structure identified through CFA;
- whereas descriptive analyses using composite variables were retained to facilitate comparison with prior organizational commitment literature using the original scoring structure.

In addition, we now clarify that:
“the removal of weak-performing items was intended to refine the operational representation of continuance commitment rather than redefine the construct itself.”

These revisions were intended to improve analytical coherence and strengthen interpretive transparency across the different stages of analysis.

4. Measurement Integrity and Model Modifications

**Comment:**
The measurement revisions are central to the manuscript’s argument, yet they are not fully justified or interpreted. Removing CC11 and CC12, and introducing correlated residuals within the distributed leadership scale, may improve model fit, but at present those decisions appear to be driven mainly by statistical convenience rather than conceptual reasoning. “That creates a problem for construct validity. The manuscript does not adequately address whether dropping the continuance commitment items changes what the construct now represents, nor does it explain how the revised version compares with the original validated scale. The same issue arises with the correlated residuals: they are introduced as a way of improving fit, but the paper does not offer a substantive explanation of what those shared residual relationships mean.”

**Response:**
We appreciate this detailed methodological critique and substantially expanded the conceptual and statistical justification for all measurement-related decisions.

Regarding the continuance commitment subscale, the revised manuscript now explains that:

- items CC11 and CC12 showed consistently weak loadings and negligible item-total correlations across analyses;
- conceptually, these items appeared to reflect perceived lack of alternatives and external constraints more than attachment-based commitment itself;
- and this distinction aligns with prior literature suggesting that continuance commitment may itself contain multiple conceptual dimensions (e.g., Meyer et al., 2002; Powell & Meyer, 2004).

We now explicitly clarify that the revised four-item version represents a narrower operationalization of continuance commitment compared with the original validated six-item scale. The manuscript also now notes that comparisons with studies using the original six-item version should therefore be interpreted cautiously.

To further evaluate continuity between the original and revised versions, a follow-up analysis demonstrated a very strong association between the original six-item composite and the revised four-item composite, suggesting substantial overlap despite the narrower operationalization.

The revised manuscript now explicitly states:
“The removal of weak-performing items was therefore intended to refine the operational representation of continuance commitment rather than redefine the construct itself.”

Regarding the distributed leadership scale:

- the rationale for correlating residuals between items 1 and 2 was expanded conceptually and statistically;
- the revised manuscript now explains that both items refer specifically to the principal’s leadership behavior and capture closely related aspects of goal-directed school management;
- therefore, beyond the shared latent factor, the items likely contain additional shared variance due to overlapping content and a common referent.

The correlated residual was therefore interpreted as a theoretically meaningful local dependence relationship rather than a purely data-driven model adjustment.

In addition, we clarified that the same correlated residual structure was also reported during the original scale development study by Özer and Beycioğlu (2013), suggesting that this overlap may represent a recurring characteristic of the scale itself rather than an artifact specific to the present sample.

**Comment:**
“The bifactor model also deserves fuller interpretation. The results seem to indicate a strong general factor, yet the manuscript continues to discuss the three commitment dimensions as though their distinctiveness were unproblematic.”

**Response:**
Thank you for this important observation. We agree that the bifactor results indicate a strong general organizational commitment factor and that this required fuller interpretation.

The revised manuscript now clarifies that although affective commitment, normative commitment, and continuance commitment remain theoretically meaningful, their empirical distinctiveness in the present sample was more limited than a simple three-factor interpretation would suggest.

Specifically:

- the high omega hierarchical coefficient for the general factor (OmegaH = 0.836) suggested that the overall organizational commitment score behaved as largely unidimensional;
- whereas the relatively low omega hierarchical subscale coefficients indicated that some subdimensions retained limited reliable variance once the general factor was taken into account.

Continuance commitment retained comparatively more specific variance, although it remained psychometrically weaker than the general factor.

Accordingly, the revised manuscript now emphasizes that:

- the structural findings are interpreted primarily at the level of overall organizational commitment;
- while the three subdimensions are interpreted with greater caution.

The revised manuscript now explicitly states:
“While the three commitment dimensions remain theoretically meaningful, their empirical distinctiveness in the present sample was more limited than a simple three-factor interpretation would imply.”

These revisions were intended to strengthen construct validity interpretation and improve conceptual transparency.

5. **Inferential Validity and Interpretation of Results**

**Comment:**
“The interpretation of the findings sometimes goes beyond…and being more explicit about what the design allows the authors to claim and what it does not.”

**Response:**
We thank the reviewer for this important methodological observation. We agree that some of the original wording may have implied stronger directional or causal inference than our cross-sectional correlational design can support.

In response, we systematically revised the interpretive language throughout the manuscript.

Specifically:

- terms such as “predicts,” “influence,” “effect,” “driver,” “enhances,” “fosters,” and “shapes” were revised where necessary and replaced with associational language such as “is associated with,” “is related to,” “shows a significant association with,” and “accounts for variance in” where appropriate.

In addition:

- the Discussion and Conclusion sections now explicitly acknowledge the limitations associated with cross-sectional self-report data;
- and additional discussion regarding common method bias, perceptual alignment, and shared method variance was added.

The revised manuscript now states:
“As the findings are based on cross-sectional self-report data collected at a single point in time, the observed relationship may partially reflect shared perceptual or method-related variance.”

The Conclusion section also now explicitly states:
“Given the cross-sectional and correlational design of this study, the findings should not be interpreted as causal relationships.”

We also clarified that SEM was used to test theoretically specified structural associations among latent variables, but does not itself establish causality.

These revisions were intended to ensure that all claims remain proportionate to the methodological limitations of the study design.

6. **Empirical Interpretation and Substantive Insight**

**Comment:**
The manuscript reported findings statistically but did not sufficiently interpret their substantive meaning.

**Response:**
We substantially expanded the Discussion section to deepen the interpretation of the findings.

The revised manuscript now:

- explains why distributed leadership may be more strongly associated with organizational commitment than school type;
- interprets the findings through relational leadership, professional interaction, and social exchange processes;
- examines why continuance commitment demonstrated a comparatively weaker relationship;
- and integrates the UAE context more directly into the interpretation of the findings.

For example, the revised discussion now explains:
“This suggests that teachers’ lived professional experiences within schools may be shaped more strongly by everyday leadership interactions than by broader institutional distinctions between public and private schools.”

The manuscript also now discusses how distributed leadership may foster professional cohesion across culturally diverse school communities and how multicultural educational contexts may shape teachers’ perceptions of leadership and commitment.

These revisions were intended to move the discussion beyond statistical reporting toward stronger substantive interpretation.

7. **Contextual Contribution (UAE Setting)**

**Comment:**
The UAE context was described but not sufficiently integrated analytically into the findings.

**Response:**
We thank the reviewer for this valuable observation. The UAE context is now integrated much more directly throughout the manuscript.

Specifically:

- the Introduction and Problem Statement now more explicitly discuss workforce diversity and the coexistence of public and private educational systems;
- the Discussion sections now interpret findings in relation to multicultural teacher populations, contextual diversity, leadership practice, and institutional variation across emirates;
- and the Conclusion now explicitly positions the UAE as a culturally diverse educational setting in which distributed leadership may hold particular relevance.

The revised manuscript also now discusses:

- differences between Emirati, Arab, and non-Arab teachers;
- contextual variation across emirates;
- and how distributed leadership may support professional cohesion in culturally diverse schools.

These revisions were intended to strengthen the contextual contribution of the study and ensure that the UAE setting functions analytically rather than descriptively.

8. **Methodological Transparency and Reporting**

**8.1 Sampling Strategy and Representativeness**

**Response:**
We revised the Participants and Sampling section to clarify:

- how schools were selected;
- how survey distribution was conducted;
- and the role of principals and school coordinators in facilitating access to teachers.

The revised manuscript now explicitly explains that participation remained voluntary and anonymous throughout the data collection process.

**8.2 Data Collection Context and Response Conditions**

**Response:**
The manuscript now more explicitly explains:

- how anonymity and voluntariness were protected;
- that participation was voluntary;
- that teachers could withdraw at any time;
- and that no identifying information was shared with school administrators.

Additional wording was added to clarify efforts to reduce potential response bias associated with school-based distribution procedures.

**8.3 Measurement Approach and Self-Report Dependence**

**Comment:**
“All of the key variables rely on perception-based measures…”

**Response:**
We addressed this concern by expanding the methodological caution and interpretive limitations associated with perception-based self-report data.

Specifically:

- the manuscript now explicitly discusses common method bias, shared perceptual variance, and perceptual alignment;
- the Discussion and Conclusion sections now acknowledge that the observed associations may partially reflect shared response tendencies associated with the use of self-report measures collected at a single point in time;
- and the limitations section now explicitly states that the design does not permit causal inference.

Future research recommendations were also expanded to encourage:

- longitudinal designs,
- multi-source data collection,
- and integration of principal-reported leadership data with teacher-reported commitment measures.

**8.4 Analytical Procedures and Assumptions**

**Response:**
The Data Analysis Strategy section was expanded to provide fuller detail regarding:

- assumption testing,
- outlier handling,
- normality evaluation,
- use of Welch’s ANOVA where homogeneity assumptions were violated,
- and fit index criteria.

These revisions were intended to strengthen analytical transparency and reproducibility.

**8.5 Model Specification and Validation Strategy**

**Comment:**
“The combined use of CFA, SEM, and split-sample validation … for judging the stability of the model.”

**Response:**
Thank you for this helpful comment. We agree that the original manuscript did not explain the role of the calibration and validation samples with sufficient clarity.

In revising the manuscript, we clarified that our use of the split-sample approach was intended primarily to examine the stability of item functioning and the consistency of measurement-related decisions across two independent subsamples.

More specifically:

- we examined how the items of each scale performed across both subsamples;
- and evaluated whether item-retention, item-removal, and theory-consistent model-refinement decisions were supported consistently across both groups.

We also clarified that our procedure was not intended as a strict calibration-first, validation-second sequence in which all modifications were identified exclusively in one sample and then tested unchanged in the other. Rather, the two subsamples were used to assess whether problematic items and theoretically defensible model refinements showed similar psychometric patterns across both groups. In this sense, the split-sample strategy served as an internal robustness check on the stability of the measurement structure.

The revised manuscript now explicitly states:
“The purpose of this split-sample approach was to examine the stability of item functioning and the consistency of measurement-related decisions across two independent subsamples.” These revisions were intended to improve transparency regarding the validation logic of the measurement procedures.

**8.6 Integration of Analytical Stages**

**Response:**
We substantially reorganized the Data Analysis Strategy section to improve the narrative flow across analytical stages.

The revised manuscript now explicitly explains:

- CFA as the measurement validation stage;
- SEM as the structural association stage;
- descriptive analyses as contextual interpretation;
- and group comparisons as demographic exploration.

These revisions were intended to strengthen overall methodological coherence.

9. **Overall Integration**

**Comment:**
The manuscript contained strong individual components but lacked sufficient integration across theory, measurement, analysis, and interpretation.

**Response:**
We sincerely appreciate this comprehensive observation and undertook substantial revisions to improve integration throughout the manuscript.

The revised manuscript now:

- defines a clearer primary contribution;
- aligns the theoretical framework more directly with the tested SEM model;
- clarifies the relationship between descriptive and latent analyses;
- provides stronger conceptual justification for measurement decisions;
- integrates the UAE context analytically rather than descriptively;
- recalibrates causal language to associational language;
- and substantially deepens the substantive interpretation of the findings.

In addition:

- the Discussion section was reorganized to align directly with the revised research questions;
- the Conclusion was rewritten to synthesize theory, methodology, context, and findings around a more unified argument;
- and transitions between analytical stages were clarified throughout the manuscript.

We believe these revisions substantially improved the conceptual clarity, methodological transparency, analytical coherence, and interpretive precision of the study.

We thank reviewer #1 for their thoughtful and constructive feedback. Their comments significantly strengthened the manuscript, and we are grateful for the opportunity to revise and improve the study.

***************************************************************************

**REVIEWER #2**

**Comment 1:**

“The manuscript’s central contribution is not yet defined sharply enough. The paper appears to move between three purposes testing the DL to OC relationship, refining measurement, and highlighting the UAE setting without clearly establishing which of these is primary.”

### **Response:**

We sincerely thank the reviewer for this important observation. In response, we substantially revised the manuscript to establish a clearer hierarchy among the study’s contributions and improve the overall conceptual coherence of the paper.

The revised manuscript now explicitly positions the association between distributed leadership and teachers’ organizational commitment as the primary contribution of the study. The measurement refinement procedures and the UAE contextual contribution are now framed as supporting dimensions that strengthen the interpretation of this central relationship rather than functioning as separate or competing aims.

To address this concern, we revised the:

- Abstract,
- Introduction,
- Problem Statement,
- Research Questions,
- Data Analysis Strategy,
- Discussion,
- and Conclusion.

The revised Introduction now frames the study more explicitly around the relationship between distributed leadership and organizational commitment, while the Discussion and Conclusion sections were reorganized to ensure that all findings are interpreted in relation to this central argument.

In particular, the discussion was restructured so that the measurement findings are treated primarily as foundational psychometric work supporting the interpretation of the structural relationship between distributed leadership and organizational commitment, rather than as an independent contribution detached from the substantive aims of the study.

The revised conclusion now explicitly states:
“This study’s primary contribution lies in explaining how distributed leadership is related to teachers’ organizational commitment in UAE schools.”

These revisions were intended to improve alignment between the study aims, theoretical framing, measurement decisions, analytical strategy, and interpretation.

## Comment 2:

“A related issue concerns how the theoretical elements are brought together. The manuscript draws on distributed leadership, the three-component model of commitment, and social exchange theory, but these strands do not yet form a fully integrated explanatory framework. The relationships you test are plausible, but the mechanisms behind them remain somewhat implicit. Making these connections more explicit especially why different dimensions of commitment respond differently would deepen the analytical foundation of the study.”

### **Response**

We appreciate this important observation and substantially revised the theoretical framework and discussion sections to strengthen theoretical integration.

The revised manuscript now explains more explicitly how distributed leadership may foster relational conditions such as trust, reciprocity, collegiality, professional inclusion, shared responsibility, and professional recognition. These mechanisms are now directly connected to affective commitment and normative commitment through Social Exchange Theory.

The manuscript now clarifies that distributed leadership is conceptualized not simply as a structural leadership arrangement, but as a relational mechanism facilitating reciprocal professional exchanges characterized by trust, recognition, and shared participation. In this framework, affective commitment and normative commitment are understood as more responsive to relational and social exchange processes, whereas continuance commitment is interpreted as more strongly associated with perceived costs of leaving, economic considerations, and external constraints.

Accordingly, the revised discussion now explains more directly why distributed leadership demonstrated stronger associations with affective and normative commitment than with continuance commitment.

These revisions were intended to ensure that the structural model appears theoretically grounded rather than empirically assembled.

## Comment 3:

“The measurement decisions are also central to your contribution, yet they would benefit from more conceptual grounding. Some of the modifications improve statistical fit, but their implications for the meaning of the constructs are not fully explored. As a reader, I found myself wanting a clearer sense of how these revised measures relate to the original scales and what that means for interpreting your findings within the broader literature.”

### **Response**

We thank the reviewer for this important methodological observation. In response, we substantially expanded the conceptual and psychometric justification for the measurement refinements introduced in the study.

Regarding continuance commitment, the revised manuscript now explains that items CC11 and CC12 consistently demonstrated weak factor loadings and negligible item-total correlations across analyses. Beyond their statistical weakness, these items also appeared conceptually to reflect perceived lack of alternatives and external constraints more than attachment-based commitment itself. This distinction aligns with prior literature suggesting that continuance commitment may contain multiple conceptual dimensions (e.g., Meyer et al., 2002; Powell & Meyer, 2004).

The manuscript now explicitly clarifies that the revised four-item continuance commitment subscale represents a narrower operationalization of continuance commitment compared with the original validated six-item scale. We also now state explicitly that comparisons with studies using the original six-item version should therefore be interpreted cautiously.

The revised manuscript now explicitly states:
“The removal of weak-performing items was therefore intended to refine the operational representation of continuance commitment rather than redefine the construct itself.”

With regard to the distributed leadership scale, we also strengthened the conceptual explanation for correlating the residuals between Items 1 and 2. The revised manuscript now explains that both items refer specifically to the principal’s leadership behavior and capture closely related aspects of goal-directed school leadership. Accordingly, beyond the shared latent factor, the items likely contain additional shared variance due to overlapping content and a common referent.

The correlated residual was therefore interpreted as a theoretically meaningful local dependence relationship rather than a purely data-driven model adjustment introduced solely to improve fit.

In addition, we clarified that this same correlated residual specification was also reported during the original development of the distributed leadership scale by Özer and Beycioğlu (2013), suggesting that the overlap may represent a recurring characteristic of the scale itself rather than an artifact of the present sample.

The bifactor interpretation was also expanded substantially. The revised manuscript now clarifies that although affective commitment, normative commitment, and continuance commitment remain theoretically meaningful, their empirical distinctiveness in the present sample was more limited than a simple three-factor interpretation would suggest.

Specifically:

- the high omega hierarchical coefficient for the general factor suggested that the overall organizational commitment score behaved as largely unidimensional;
- whereas the relatively low omega hierarchical subscale coefficients indicated that some subdimensions retained limited reliable variance once the general factor was taken into account.

Accordingly, the revised manuscript now emphasizes that the structural findings are interpreted primarily at the level of overall organizational commitment, while the three subdimensions are interpreted with greater caution.

## Comment 4:

“In terms of interpretation, the results are clearly presented, and the strength of the relationships is striking. However, the discussion would benefit from a more reflective engagement with these patterns. Why does distributed leadership appear so dominant? What might explain the comparatively weaker role of continuance commitment? There is an opportunity here to move beyond reporting toward explanation especially by drawing more directly on the contextual features of the UAE that you introduce earlier in the manuscript.”

### **Response**

We appreciate this thoughtful suggestion and substantially expanded the Discussion section to deepen the substantive interpretation of the findings.

The revised discussion now examines:

- why distributed leadership may be more strongly associated with organizational commitment than school type;
- why continuance commitment demonstrated comparatively weaker associations;
- and how the multicultural educational context of the UAE may shape teachers’ perceptions of leadership and commitment.

The manuscript now discusses how distributed leadership may foster inclusion, shared professional understanding, collegial trust, and professional cohesion across culturally diverse school environments. We also expanded the interpretation of continuance commitment by explaining that this dimension is conceptually less connected to relational leadership processes because it is more strongly associated with perceived costs of leaving, contractual considerations, and external constraints.

In addition, the revised manuscript now discusses how teachers’ lived professional experiences may be shaped more strongly by everyday leadership interactions and shared professional practices than by broader institutional distinctions between public and private schools.

The UAE context was also integrated more directly throughout the discussion sections. The revised manuscript now interprets the findings in relation to multicultural teacher populations, contextual diversity across emirates, and the coexistence of public and private educational systems within the UAE.

These revisions were intended to move the discussion beyond statistical reporting toward stronger contextual and theoretical explanation.

## Comment 5:

“I also encourage a slight recalibration of the language used to describe the findings. Given the cross-sectional and perception-based nature of the data, framing the results in associational rather than causal terms would better align the claims with the design. This does not diminish the value of the findings; rather, it strengthens their credibility.”

### **Response**

We thank the reviewer for this important methodological observation. We agree that some wording in the original manuscript may have implied stronger directional or causal inference than our cross-sectional correlational design can support.

In response, we systematically revised the manuscript to align the interpretive language more closely with the evidentiary scope of the study.

Specifically, terms such as:

- “predicts,”
- “influences,”
- “effects,”
- “drivers,”
- “enhances,”
- “fosters,”
- and “shapes”

were revised where appropriate and replaced with associational language such as:

- “is associated with,”
- “is related to,”
- “shows a significant association with,”
- and “accounts for variance in.”

In addition, the Discussion and Conclusion sections now explicitly acknowledge the limitations associated with cross-sectional self-report data and the possibility of shared perceptual or method-related variance.

The revised manuscript now explicitly states:
“As the findings are based on cross-sectional self-report data collected at a single point in time, the observed relationship may partially reflect shared perceptual or method-related variance.”

The Conclusion section now also states:
“Given the cross-sectional and correlational design of this study, the findings should not be interpreted as causal relationships.”

We also clarified that although SEM allows the examination of theoretically specified structural associations among latent variables, it does not itself establish causality or temporal ordering.

These revisions were intended to strengthen methodological precision and ensure that all interpretations remain proportionate to the study design.

## Comment 6:

“Overall, the manuscript contains many of the elements of a strong contribution. What it needs now is greater alignment between its aims, theoretical framing, measurement choices, analytical strategy, and interpretation. With careful revision, these components can be brought together into a more coherent and persuasive whole.”

### **Response**

We sincerely appreciate this comprehensive observation and treated it as a guiding principle throughout the revision process.

In response, we substantially revised the manuscript to improve alignment across:

- the study aims,
- theoretical framework,
- measurement decisions,
- analytical procedures,
- interpretation of findings,
- and contextual contribution.

The revised manuscript now:

- defines a clearer primary contribution centered on the relationship between distributed leadership and organizational commitment;
- integrates Social Exchange Theory more explicitly into the explanation of the findings;
- clarifies the conceptual basis for the measurement refinements;
- explains the analytical logic linking CFA, SEM, descriptive analyses, and demographic comparisons;
- recalibrates causal language to associational language;
- and strengthens the interpretation of findings within the multicultural UAE educational context.

The Discussion section was also reorganized to align more directly with the revised research questions and to improve the logical progression from measurement findings to structural interpretation and contextual explanation.

We are grateful for the reviewer’s thoughtful and constructive feedback, which significantly strengthened the conceptual coherence, methodological transparency, and interpretive clarity of the manuscript.
