# Supplementary material for: Association between distributed leadership and teachers’ organizational commitment: Evidence from public and private schools in the United Arab Emirates
Source: PLoS One. 2026 Jun 23;21(6):e0351540. doi: 10.1371/journal.pone.0351540 (PMC13289934; doi:10.1371/journal.pone.0351540)
Supplement: Peer review history

# Peer review: Decision Letter 0

**DOI:** 10.1371/journal.pone.0351540.r001

**Authors:** Yaser Mohammed Al-Worafi

4 Apr 2026

PONE-D-26-09053

Distributed Leadership as a Predictor of Teachers' Organizational Commitment: Evidence from Public and Private Schools in the United Arab Emirates

PLOS One

Dear Dr. Ibrahim,

Thank you for submitting your manuscript to PLOS ONE. After careful consideration, we feel that it has merit but does not fully meet PLOS ONE’s publication criteria as it currently stands. Therefore, we invite you to submit a revised version of the manuscript that addresses the points raised during the review process.

Please submit your revised manuscript by May 19 2026 11:59PM. If you will need more time than this to complete your revisions, please reply to this message or contact the journal office at plosone@plos.org. When you're ready to submit your revision, log on to https://www.editorialmanager.com/pone/ and select the 'Submissions Needing Revision' folder to locate your manuscript file.

Please include the following items when submitting your revised manuscript:

- A letter that responds to each point raised by the academic editor and reviewer(s). You should upload this letter as a separate file labeled 'Response to Reviewers'.
- A marked-up copy of your manuscript that highlights changes made to the original version. You should upload this as a separate file labeled 'Revised Manuscript with Track Changes'.
- An unmarked version of your revised paper without tracked changes. You should upload this as a separate file labeled 'Manuscript'.

If you would like to make changes to your financial disclosure, please include your updated statement in your cover letter. Guidelines for resubmitting your figure files are available below the reviewer comments at the end of this letter.

If applicable, we recommend that you deposit your laboratory protocols in protocols.io to enhance the reproducibility of your results. Protocols.io assigns your protocol its own identifier (DOI) so that it can be cited independently in the future. For instructions see: https://journals.plos.org/plosone/s/submission-guidelines#loc-laboratory-protocols. Additionally, PLOS ONE offers an option for publishing peer-reviewed Lab Protocol articles, which describe protocols hosted on protocols.io. Read more information on sharing protocols at https://plos.org/protocols?utm_medium=editorial-email&utm_source=authorletters&utm_campaign=protocols.

As the corresponding author, your ORCID iD is verified in the submission system and will appear in the published article. PLOS supports the use of ORCID, and we encourage all coauthors to register for an ORCID iD and use it as well. Please encourage your coauthors to verify their ORCID iD within the submission system before final acceptance, as unverified ORCID iDs will not appear in the published article. *Only*   the individual author can complete the verification step; PLOS staff *cannot*   verify ORCID iDs on behalf of authors.

We look forward to receiving your revised manuscript.

Kind regards,

Yaser Mohammed Al-Worafi

Academic Editor

PLOS One

Journal Requirements:

When submitting your revision, we need you to address these additional requirements.

1.Please ensure that your manuscript meets PLOS ONE's style requirements, including those for file naming. The PLOS ONE style templates can be found at

https://journals.plos.org/plosone/s/file?id=wjVg/PLOSOne_formatting_sample_main_body.pdf and

https://journals.plos.org/plosone/s/file?id=ba62/PLOSOne_formatting_sample_title_authors_affiliations.pdf

2. We note that there is identifying data in the Supporting Information file “Distributed Leadership Data.xlsx” Due to the inclusion of these potentially identifying data, we have removed this file from your file inventory. Prior to sharing human research participant data, authors should consult with an ethics committee to ensure data are shared in accordance with participant consent and all applicable local laws.

Data sharing should never compromise participant privacy. It is therefore not appropriate to publicly share personally identifiable data on human research participants. The following are examples of data that should not be shared:

-Name, initials, physical address

-Ages more specific than whole numbers

-Internet protocol (IP) address

-Specific dates (birth dates, death dates, examination dates, etc.)

-Contact information such as phone number or email address

-Location data

-ID numbers that seem specific (long numbers, include initials, titled “Hospital ID”) rather than random (small numbers in numerical order)

Data that are not directly identifying may also be inappropriate to share, as in combination they can become identifying. For example, data collected from a small group of participants, vulnerable populations, or private groups should not be shared if they involve indirect identifiers (such as sex, ethnicity, location, etc.) that may risk the identification of study participants.

Additional guidance on preparing raw data for publication can be found in our Data Policy (https://journals.plos.org/plosone/s/data-availability#loc-human-research-participant-data-and-other-sensitive-data) and in the following article: http://www.bmj.com/content/340/bmj.c181.long.

Please remove or anonymize all personal information (<specific identifying information in file to be removed>), ensure that the data shared are in accordance with participant consent, and re-upload a fully anonymized data set. Please note that spreadsheet columns with personal information must be removed and not hidden as all hidden columns will appear in the published file.

3. Please amend your authorship list in your manuscript file to include author “Ali Ibrahim, Shaikha Alshehhi and Nikolaos Tsingilis”

4. Please amend your list of authors on the manuscript to ensure that each author is linked to an affiliation. Authors’ affiliations should reflect the institution where the work was done (if authors moved subsequently, you can also list the new affiliation stating “current affiliation:….” as necessary).

5. Please include captions for your Supporting Information files at the end of your manuscript, and update any in-text citations to match accordingly. Please see our Supporting Information guidelines for more information: http://journals.plos.org/plosone/s/supporting-information.

6. If the reviewer comments include a recommendation to cite specific previously published works, please review and evaluate these publications to determine whether they are relevant and should be cited. There is no requirement to cite these works unless the editor has indicated otherwise.

[Note: HTML markup is below. Please do not edit.]

Reviewer's Responses to Questions

**Comments to the Author**

1. Is the manuscript technically sound, and do the data support the conclusions?

The manuscript must describe a technically sound piece of scientific research with data that supports the conclusions. Experiments must have been conducted rigorously, with appropriate controls, replication, and sample sizes. The conclusions must be drawn appropriately based on the data presented.

Reviewer #1: Partly

Reviewer #2: Yes

**********

2. Has the statistical analysis been performed appropriately and rigorously?

Reviewer #1: Yes

Reviewer #2: Yes

**********

3. Have the authors made all data underlying the findings in their manuscript fully available?

The PLOS Data policy requires authors to make all data underlying the findings described in their manuscript fully available without restriction, with rare exception (please refer to the Data Availability Statement in the manuscript PDF file). The data should be provided as part of the manuscript or its supporting information, or deposited to a public repository. For example, in addition to summary statistics, the data points behind means, medians and variance measures should be available. If there are restrictions on publicly sharing data—e.g. participant privacy or use of data from a third party—those must be specified.

Reviewer #1: No

Reviewer #2: Yes

**********

4. Is the manuscript presented in an intelligible fashion and written in standard English?

PLOS ONE does not copyedit accepted manuscripts, so the language in submitted articles must be clear, correct, and unambiguous. Any typographical or grammatical errors should be corrected at revision, so please note any specific errors here.

Reviewer #1: Yes

Reviewer #2: Yes

**********

5. Review Comments to the Author

Please use the space provided to explain your answers to the questions above. You may also include additional comments for the author, including concerns about dual publication, research ethics, or publication ethics. (Please upload your review as an attachment if it exceeds 20,000 characters)

Reviewer #1: This study is mainly a methodological exercise in linking distributed leadership to various forms of organizational commitment. The statistics and latent construct modeling are all according to the standards and not in themselves problematic as the analyses are what they are. But, conversely, nor do they merit publication in themselves. I am a great fan of conducting analyses using data from non-WEIRD countries, but there need to be theoretically meaningful questions asked that then motivate the types of analyses performed.

What I find underdeveloped in this article is the theoretical purpose and line of argumentation in the article. All the statistics in the world do not help if there are no clear questions to be argued, asked, answered and discussed. When I enter the terms "organizational commitment" and "distributed leadership" in Google Scholar, I get about 8,800 results immediately so there is no lack of previous research in this field.

That said, I do agree with the authors that the UAE could make an interesting context for repeating some of these research topics but in that case, I would want to have more specific research questions and a more pointed analysis with discussion. It is quite possible, as the authors say, that there has not been enough cross-cultural studies in this field but the problem reappears in the catalogue of research questions: What is particular to the UAE, what are the reasons to expect differences from other places, and how do these considerations bear on the actual analyses performed and the subsequent findings? At the moment, I read the introduction and the theoretical part more as an evolving list of this and that, spanning from the possible mechanisms and factor structures in the broader theoretical discussions to local industrial and cultural impacts. Is this about score levels, construct validation, instrument calibration or about describing emergent features of the educational system in the UAE? The results section contains quite detailed sections on modifying the models, deleting and keeping items and lists of tables with statistical results that to me become unreadable because I am drowning in information that I cannot relate to particular research quesions. For example, what should I as a reader think about the difference in statistics between Ras Al Khaimah, Fujairah and Sharjah if there are no theoretical or empirical preparations to use these differences to test hypotheses or assumptions? When we then get to the discussion section, many of these analyses and tables seem underutilized. The reader is told that "DL, as perceived by teachers, was widely adopted across public and private schools" with some numbers showing more or less deviations from this but I am still wondering about questions like: Yes, statistical differences, but do they matter? What do they reflect? Why do UAE citizens display different levels of DL than others, was this an interesting, expected finding or accidental? Since the introduction and theoretical part do not prepare more that what seems like a catalogue of quite unrelated questions (e.g., 1. do principals practice DL and 4) do levels of OC and its subcomponents (AC, NC, CC) significantly differ according to demographic characteristics), I find it hard to judge whether the analyses bring new knowledge about DL, commitment, cross-cultural leadership research or specific knowledge about what happens in the educational sector in the UAE.

As the article reads presently, without more incisive theoretical specifications, it could have been summed up in a few poignant tables about score levels and correlations related to the various variables of interest. An improved theoretical framework or a more compact table of salient findings would be all the more important given that the present dataset is a cross-sectional, same method sample with very weak design for extracting mechanisms other that correlations.

And, as a final aside, the authors have diligently enough provided the dataset, but not the item texts. Many of the analyses performed here relating to latent modeling are now possible to perform a priori and it would have been much more interesting to read about how expected values of latent models are actually deviating from observed response patterns in the UAE (see e.g., Pillet, J.-C., Larsen, K. R., Dobolyi, D., Queiroz, M., Handler, A., Arnulf, J. K., & Sharma, R. (2025). AI-Augmented Content Validation in Behavioral Research: Development and Evaluation of the RATER System. Management Information Systems Quarterly, 1-33. https://doi.org/10.25300/misq/2025/18946).

I sum, I think this article contains an interesting dataset and timely cross-cultural observations but the manuscript as it reads now is conceptually and theoretically underdeveloped and does not merit publication in its present form.

Reviewer #2: Dear Author,

Thank you for the opportunity to read and engage with your manuscript. It is clear that a considerable amount of effort has gone into assembling a substantial dataset and applying a range of analytical techniques to a topic that remains highly relevant within educational leadership research. The study has real potential, particularly in its attempt to connect distributed leadership with organizational commitment within the UAE context.

The main issue at present is that the manuscript’s central contribution is not yet defined sharply enough. The paper appears to move between three purposes testing the DL to OC relationship, refining measurement, and highlighting the UAE setting without clearly establishing which of these is primary.

A related issue concerns how the theoretical elements are brought together. The manuscript draws on distributed leadership, the three-component model of commitment, and social exchange theory, but these strands do not yet form a fully integrated explanatory framework. The relationships you test are plausible, but the mechanisms behind them remain somewhat implicit. Making these connections more explicit especially why different dimensions of commitment respond differently would deepen the analytical foundation of the study.

The measurement decisions are also central to your contribution, yet they would benefit from more conceptual grounding. Some of the modifications improve statistical fit, but their implications for the meaning of the constructs are not fully explored. As a reader, I found myself wanting a clearer sense of how these revised measures relate to the original scales and what that means for interpreting your findings within the broader literature.

In terms of interpretation, the results are clearly presented, and the strength of the relationships is striking. However, the discussion would benefit from a more reflective engagement with these patterns. Why does distributed leadership appear so dominant? What might explain the comparatively weaker role of continuance commitment? There is an opportunity here to move beyond reporting toward explanation especially by drawing more directly on the contextual features of the UAE that you introduce earlier in the manuscript.

I also encourage a slight recalibration of the language used to describe the findings. Given the cross-sectional and perception-based nature of the data, framing the results in associational rather than causal terms would better align the claims with the design. This does not diminish the value of the findings; rather, it strengthens their credibility.

Overall, the manuscript contains many of the elements of a strong contribution. What it needs now is greater alignment between its aims, theoretical framing, measurement choices, analytical strategy, and interpretation. With careful revision, these components can be brought together into a more coherent and persuasive whole.

I hope these comments are helpful as you continue to develop the manuscript. There is a solid foundation here, and with refinement, the study can make a meaningful contribution to the field.

Warm regards,

COMMENTS

1. Core Contribution and Conceptual Framing

The manuscript takes up a worthwhile and timely topic, but its central contribution is still difficult to locate. As written, the paper seems to be trying to do three things at once: examine the relationship between distributed leadership and organizational commitment, refine the measurement of continuance commitment, and contribute evidence from the UAE context. Any one of these could support a meaningful scholarly contribution. The difficulty is that the manuscript does not yet make clear which of them is doing the main intellectual work, or how they fit together within a single, well-defined argument.

That lack of hierarchy weakens the framing of the study. In some places, the paper reads as a confirmatory test of an established relationship. In others, it moves toward measurement refinement. Elsewhere, it presents itself as a contextually distinctive contribution grounded in the UAE setting. Because these strands are not clearly ordered, the paper feels somewhat split in its purpose, and the reader is left uncertain about what the manuscript most wants to add to the literature.

The paper would become more persuasive if it identified one primary contribution and treated the remaining elements as supporting dimensions of that contribution. If the central aim is to explain the DL–OC relationship, then the scale refinements and the UAE context should be positioned in service of that broader question. If, instead, the real contribution lies in measurement, then the revision of the continuance commitment scale needs to move much closer to the center of the paper. Making that decision would sharpen the introduction, strengthen the interpretation of the findings, and give the manuscript a clearer sense of significance.

2. Theoretical Coherence and Model Justification

The manuscript brings together distributed leadership, the Three-Component Model of organizational commitment, and Social Exchange Theory, but these elements are not yet woven into a framework that fully explains the structural model being tested. The ingredients are present, yet the theoretical logic that connects them remains underdeveloped.

The discussion of the links between distributed leadership and the three dimensions of commitment stays fairly general. The manuscript suggests that these relationships exist, but it does not sufficiently explain the mechanisms through which they are expected to emerge. That leaves the model looking more empirically assembled than theoretically generated.

This is especially noticeable in the treatment of affective, normative, and continuance commitment. The manuscript states that distributed leadership should relate differently to each dimension, but those differences are not really derived from theory. Social Exchange Theory is invoked, though its role remains mostly implicit, and the reader is not shown clearly how it informs the specific paths estimated in the SEM.

The theoretical framing would be much stronger if these links were made more explicit. The manuscript could, for instance, explain how distributed leadership fosters relational conditions such as trust, reciprocity, and shared responsibility, which in turn are more likely to nourish affective and normative commitment than continuance commitment. A more direct alignment between theory and model specification would deepen the explanatory force of the paper and make the structural analysis feel more fully justified.

3. Analytical Design and Internal Consistency

The manuscript moves through several analytical stages—descriptive comparison, measurement refinement, and structural modeling—but the relationship among these stages is not yet fully clear. In particular, the paper relies on the full scales for descriptive analyses and then shifts to modified scales, after item removal and model adjustment, for the SEM. That transition is important, but it is not explained in enough depth.

As a result, the study develops an internal inconsistency in the way its key constructs are operationalized. It becomes difficult to tell whether the structural findings should be read as evidence about the original constructs or about revised versions of those constructs. That ambiguity matters because it affects how the results can be compared across research questions and across different parts of the paper.

The manuscript would benefit from a clearer explanation of the analytical logic that connects these phases. The authors should spell out why different versions of the measures were used at different stages, whether the SEM results depend on a reconceptualization of the constructs, and how readers should understand the relationship between the descriptive and structural findings. A more explicit account of that progression would make the analysis feel more coherent and easier to interpret.

4. Measurement Integrity and Model Modifications

The measurement revisions are central to the manuscript’s argument, yet they are not fully justified or interpreted. Removing CC11 and CC12, and introducing correlated residuals within the distributed leadership scale, may improve model fit, but at present those decisions appear to be driven mainly by statistical convenience rather than conceptual reasoning.

That creates a problem for construct validity. The manuscript does not adequately address whether dropping the continuance commitment items changes what the construct now represents, nor does it explain how the revised version compares with the original validated scale. The same issue arises with the correlated residuals: they are introduced as a way of improving fit, but the paper does not offer a substantive explanation of what those shared residual relationships mean. Without that explanation, it is difficult to know whether the changes reflect theoretically meaningful overlap or simply model-specific adjustment.

The bifactor model also deserves fuller interpretation. The results seem to indicate a strong general factor, yet the manuscript continues to discuss the three commitment dimensions as though their distinctiveness were unproblematic. That tension is important and should be addressed directly.

This section would be stronger if the manuscript offered a conceptually grounded rationale for each measurement decision and then discussed the implications of those decisions more openly. The paper needs to clarify whether the revised scales still represent the same constructs, how they should be interpreted in relation to prior studies, and whether the bifactor results ultimately support a multidimensional or more largely unidimensional understanding of organizational commitment.

5. Inferential Validity and Interpretation of Results

The interpretation of the findings sometimes goes beyond what the design can reasonably support. The manuscript repeatedly uses terms such as “predicts” and “influence,” which suggest directional or causal inference, even though the data are cross-sectional and based on perceptions collected at a single point in time. SEM does not, by itself, remove that limitation, and the current wording risks overstating the evidentiary strength of the study.

The reported relationship between distributed leadership and organizational commitment is also notably strong, yet the manuscript does not examine that strength with sufficient caution. Because both constructs are measured through self-report in the same instrument, some of the observed association may reflect shared method variance or perceptual alignment rather than a purely substantive relationship.

The discussion would be more convincing if the manuscript adopted more careful, associational language and engaged more directly with the magnitude of the observed relationships. This means acknowledging the possibility of common method bias, considering whether the strength of the association may be somewhat inflated, and being more explicit about what the design allows the authors to claim and what it does not.

6. Empirical Interpretation and Substantive Insight

The results are reported with statistical detail, but the manuscript does not yet extract enough substantive meaning from them. The paper shows a strong association between distributed leadership and organizational commitment and a much weaker role for school type, yet these patterns are largely described rather than interpreted.

What is missing is a deeper explanation of why leadership perceptions seem to matter so much more than structural characteristics such as school type. That pattern could reflect something important about the lived organizational environment of schools, the salience of leadership in shaping professional experience, or the nature of the measures themselves. At present, however, the manuscript does not pause long enough to consider those possibilities.

A similar issue appears in the treatment of continuance commitment. Its weaker performance is acknowledged, but not really unpacked, even though the paper itself recognizes earlier that continuance commitment is often conceptually unstable. That leaves a potentially interesting finding underdeveloped.

The manuscript would gain depth by moving beyond statistical reporting toward fuller substantive interpretation. The discussion should explain why some relationships are especially strong, why others are limited, what these patterns imply for leadership and commitment in practice, and how they speak back to the literature rather than simply sit alongside it.

7. Contextual Contribution (UAE Setting)

The UAE is presented as an important and distinctive setting, but the manuscript does not yet make full analytical use of that context. At the moment, the setting is described more than it is interpreted.

In its current form, the paper shows that the DL–OC relationship is present in the UAE, but it does not go far enough in explaining whether the UAE context changes the meaning, strength, or implications of that relationship. Given the multicultural composition of the teaching workforce and the coexistence of public and private school systems, the context has the potential to generate genuinely useful insight. That potential, however, is only partially realized.

The contextual contribution would become much stronger if the discussion connected the findings more directly to features of the UAE educational landscape. For example, the manuscript could consider how workforce diversity, institutional arrangements, governance structures, or school sector differences shape leadership practice and commitment patterns, and whether the findings confirm or complicate what has been reported in other settings.

8. Methodological Transparency and Reporting

8.1 Sampling Strategy and Representativeness

The manuscript describes the sampling design as a combination of random and stratified sampling, but the actual recruitment process appears more mediated than that description suggests, since survey distribution was conducted through principals and school coordinators. The relationship between the intended design and the implemented procedure therefore needs clearer explanation.

A more transparent account of how participants were reached, how evenly the sample was distributed across the stated strata, and whether any selection effects may have emerged would strengthen confidence in the representativeness of the sample.

8.2 Data Collection Context and Response Conditions

The ethical procedures are clearly stated, but the manuscript says less about the practical context in which the survey responses were collected. Because the surveys were distributed through school leadership, some participants may have felt an implicit expectation to take part, or may have shaped their responses with that context in mind.

It would help to clarify more explicitly how anonymity and voluntariness were protected in practice, and whether any steps were taken to reduce the possibility of response bias in a school-based administrative setting.

8.3 Measurement Approach and Self-Report Dependence

All of the key variables—distributed leadership and organizational commitment—are measured through self-report at a single point in time. That design choice raises the possibility of common method bias and perceptual alignment, especially given the strength of the relationships reported.

The manuscript would be more methodologically balanced if it acknowledged this limitation directly and reflected on how the use of perception-based measures shapes the interpretation of the findings.

8.4 Analytical Procedures and Assumptions

The manuscript justifies the use of parametric tests on the basis of sample size, but the reporting of assumptions such as normality, homogeneity, and outlier handling remains relatively thin. Some procedures are mentioned, yet not in enough detail for the reader to fully judge analytical robustness.

Providing clearer information about assumption testing, the criteria used for outlier removal, and the handling of any departures from assumptions would improve transparency and reproducibility.

8.5 Model Specification and Validation Strategy

The combined use of CFA, SEM, and split-sample validation is one of the study’s stronger methodological features. Even so, the manuscript would benefit from clearer reporting of how decisions were made across the calibration and validation samples, especially in relation to item removal and model modification.

The paper should clarify whether modifications were identified in the calibration sample first and then tested in the validation sample, since that sequence matters for judging the stability of the model.

8.6 Integration of Analytical Stages

The manuscript uses a range of analytical techniques, including descriptive statistics, ANOVA, regression, and SEM, but the transitions between these stages are not always well explained. The move from more descriptive or exploratory analysis to confirmatory modeling would be easier to follow if the paper made clearer how each analytical stage prepares the ground for the next.

A stronger narrative thread across these stages would improve the overall methodological coherence of the manuscript.

9. Overall Integration

The manuscript has several genuine strengths in terms of dataset quality, topical relevance, and analytical ambition. At present, however, those strengths do not fully come together because the major components of the study are not yet aligned as tightly as they could be. The theoretical framing, measurement decisions, analytical strategy, and interpretation of findings sometimes seem to move alongside one another rather than reinforcing a single, unified argument.

This is why the paper can feel fragmented even though it contains many of the elements of a strong study. It is not that the manuscript lacks substance; rather, the substance is not yet fully coordinated.

The paper would be significantly stronger if it tightened the alignment across the full research process. That means ensuring that the theoretical framework genuinely supports the tested model, clarifying how measurement decisions reshape the constructs under analysis, organizing the analytical stages so that each one builds logically on the previous one, and calibrating the interpretation of the findings to the limits of the design and data.

With that level of integration, the manuscript could move from being a technically capable study with several promising parts to a more coherent and compelling scholarly contribution.

10. Reference Quality and Citation

**SEE MORE DETAILS ABOUT **REFS** IN THE ATTACHMENT**

Final Recommendation: Major Revision

The manuscript is built on a substantial empirical dataset, uses appropriate statistical techniques—including CFA and SEM with cross-validation—and addresses a topic that is clearly relevant within educational leadership research. Those features give the study a strong foundation and suggest that it has real potential to make a useful contribution to the literature. In its current form, however, the manuscript has not yet reached the level of conceptual clarity, analytical coherence, and interpretive precision needed for publication.

The encouraging part is that these are remediable problems. The manuscript would improve substantially if it defined a clear primary contribution and aligned the paper around it, strengthened the connection between theory and model specification, offered more transparent and conceptually grounded justification for its measurement decisions, and recalibrated its claims so they remain proportionate to the design. With that level of revision, the study could become not only technically sound but also intellectually persuasive. At this stage, though, major revision is the appropriate recommendation.

**********

6. PLOS authors have the option to publish the peer review history of their article (what does this mean?). If published, this will include your full peer review and any attached files.

If you choose “no”, your identity will remain anonymous but your review may still be made public.

**Do you want your identity to be public for this peer review?**   For information about this choice, including consent withdrawal, please see our Privacy Policy.

Reviewer #1: **Yes:**  Jan Ketil Arnulf

Reviewer #2: No

**********

[NOTE: If reviewer comments were submitted as an attachment file, they will be attached to this email and accessible via the submission site. Please log into your account, locate the manuscript record, and check for the action link "View Attachments". If this link does not appear, there are no attachment files.]

To ensure your figures meet our technical requirements, please review our figure guidelines: https://journals.plos.org/plosone/s/figures

You may also use PLOS’s free figure tool, NAAS, to help you prepare publication quality figures: https://journals.plos.org/plosone/s/figures#loc-tools-for-figure-preparation.

NAAS will assess whether your figures meet our technical requirements by comparing each figure against our figure specifications.

Attachment Submitted filename: REVIEW-MANUSCRIPT-PONE-D-26-09053.pdf

# Author comment: Author response to Decision Letter 1

**DOI:** 10.1371/journal.pone.0351540.r002

23 May 2026

Response to Reviewers

We sincerely thank the reviewers for their careful evaluation of our manuscript entitled “Distributed Leadership as a Predictor of Teachers' Organizational Commitment: Evidence from Public and Private Schools in the United Arab Emirates” now retitled “Association between distributed leadership and teachers' organizational commitment: Evidence from public and private schools in the United Arab Emirates.” We are grateful for the thoughtful and constructive feedback, which significantly strengthened the manuscript conceptually, theoretically, methodologically, and analytically.

In response to the comments, we undertook a substantial revision of the manuscript. The revised version now presents a clearer and more coherent central contribution focused on the association between distributed leadership (DL) and teachers’ organizational commitment (OC), while positioning the measurement refinement procedures and the UAE contextual contribution as supporting dimensions of that broader aim. We also strengthened the theoretical integration between distributed leadership, the Three-Component Model of organizational commitment, and Social Exchange Theory; clarified the analytical logic connecting CFA, SEM, descriptive analyses, and group comparisons; expanded the conceptual justification for measurement decisions; recalibrated causal language to associational language; and deepened the substantive interpretation of findings within the multicultural educational context of the UAE.

Below, we provide a detailed point-by-point response to each comment.

REVIEWER #1 COMMENTS AND RESPONSES

1. Core Contribution and Conceptual Framing

Comment:

The manuscript appeared to pursue multiple overlapping purposes simultaneously, including examining the DL–OC relationship, refining measurement, and contributing UAE evidence, without clearly identifying the primary contribution.

Response:

We appreciate this important observation and substantially revised the manuscript to establish a clearer hierarchy among the study’s contributions.

The revised manuscript now explicitly positions the association between distributed leadership and teachers’ organizational commitment as the primary contribution of the study. The measurement refinement procedures and the UAE contextual contribution are now framed as supporting dimensions that strengthen the interpretation of this central relationship rather than functioning as independent research agendas.

To address this concern, we revised the:

• Abstract,

• Introduction,

• Problem Statement,

• Research Questions,

• Data Analysis Strategy,

• Discussion,

• and Conclusion.

The Introduction now explicitly frames the study around the DL–OC relationship, while the Discussion and Conclusion sections were reorganized to ensure that all findings are interpreted in relation to this central argument.

For example, the revised conclusion now opens with:

“This study’s primary contribution lies in explaining how distributed leadership is related to teachers’ organizational commitment in UAE schools.”

These revisions were intended to improve conceptual coherence and align the theoretical framing, analytical procedures, and interpretation around a single unified scholarly contribution.

2. Theoretical Coherence and Model Justification

Comment:

The theoretical framework did not sufficiently integrate distributed leadership, the Three-Component Model, and Social Exchange Theory into a coherent explanatory framework.

Response:

We thank the reviewer for this important observation and substantially revised the theoretical framework and discussion sections to strengthen theoretical integration.

Specifically:

• the manuscript now explains more explicitly how distributed leadership may foster relational conditions such as trust, reciprocity, shared responsibility, collegiality, inclusion, and professional recognition;

• these mechanisms are now directly linked to affective commitment and normative commitment through Social Exchange Theory;

• and the weaker relationship with continuance commitment is now interpreted as conceptually expected because continuance commitment is more strongly associated with perceived costs of leaving and structural considerations rather than relational exchange processes.

The revised manuscript now explicitly explains:

“Distributed leadership is conceptualized as a relational mechanism that facilitates reciprocal exchanges characterized by trust, recognition, and shared professional responsibility.”

The SEM discussion was also revised to explain why distributed leadership appears more strongly associated with affective and normative commitment than with continuance commitment. These revisions were intended to ensure that the structural model appears theoretically generated rather than empirically assembled.

3. Analytical Design and Internal Consistency

Comment:

“The manuscript moves through several analytical stages…would make the analysis feel more coherent and easier to interpret.”

Response:

We thank the reviewer for pointing out this issue. We agree that the previous version of the manuscript was not sufficiently clear. In response, we restructured both the Data Analysis Strategy and Results sections to improve the coherence and interpretability of the analytical sequence.

Specifically:

• the revised manuscript now begins with Confirmatory Factor Analysis (CFA) to examine the underlying structure and psychometric adequacy of the variables under study;

• this is followed by Structural Equation Modeling (SEM) examining the associations between distributed leadership and organizational commitment;

• and finally descriptive and group comparison analyses are presented to explore demographic and contextual variation.

We also clarified the relationship between the descriptive analyses and the latent structural analyses. The revised manuscript now explicitly explains that:

• CFA was used to establish the measurement structure and evaluate the adequacy of the scales;

• SEM relied on the refined latent structure identified through CFA;

• whereas descriptive analyses using composite variables were retained to facilitate comparison with prior organizational commitment literature using the original scoring structure.

In addition, we now clarify that:

“the removal of weak-performing items was intended to refine the operational representation of continuance commitment rather than redefine the construct itself.”

These revisions were intended to improve analytical coherence and strengthen interpretive transparency across the different stages of analysis.

4. Measurement Integrity and Model Modifications

4.1 Comment:

The measurement revisions are central to the manuscript’s argument, yet they are not fully justified or interpreted. Removing CC11 and CC12, and introducing correlated residuals within the distributed leadership scale, may improve model fit, but at present those decisions appear to be driven mainly by statistical convenience rather than conceptual reasoning.

4.2 Comment:

“That creates a problem for construct validity. The manuscript does not adequately address whether dropping the continuance commitment items changes what the construct now represents, nor does it explain how the revised version compares with the original validated scale. The same issue arises with the correlated residuals: they are introduced as a way of improving fit, but the paper does not offer a substantive explanation of what those shared residual relationships mean.”

Response:

We appreciate this detailed methodological critique and substantially expanded the conceptual and statistical justification for all measurement-related decisions.

Regarding the continuance commitment subscale, the revised manuscript now explains that:

• items CC11 and CC12 showed consistently weak loadings and negligible item-total correlations across analyses;

• conceptually, these items appeared to reflect perceived lack of alternatives and external constraints more than attachment-based commitment itself;

• and this distinction aligns with prior literature suggesting that continuance commitment may itself contain multiple conceptual dimensions (e.g., Meyer et al., 2002; Powell & Meyer, 2004).

We now explicitly clarify that the revised four-item version represents a narrower operationalization of continuance commitment compared with the original validated six-item scale. The manuscript also now notes that comparisons with studies using the original six-item version should therefore be interpreted cautiously.

To further evaluate continuity between the original and revised versions, a follow-up analysis demonstrated a very strong association between the original six-item composite and the revised four-item composite, suggesting substantial overlap despite the narrower operationalization.

The revised manuscript now explicitly states:

“The removal of weak-performing items was therefore intended to refine the operational representation of continuance commitment rather than redefine the construct itself.”

Regarding the distributed leadership scale:

• the rationale for correlating residuals between items 1 and 2 was expanded conceptually and statistically;

• the revised manuscript now explains that both items refer specifically to the principal’s leadership behavior and capture closely related aspects of goal-directed school management;

• therefore, beyond the shared latent factor, the items likely contain additional shared variance due to overlapping content and a common referent.

The correlated residual was therefore interpreted as a theoretically meaningful local dependence relationship rather than a purely data-driven model adjustment.

In addition, we clarified that the same correlated residual structure was also reported during the original scale development study by Özer and Beycioğlu (2013), suggesting that this overlap may represent a recurring characteristic of the scale itself rather than an artifact specific to the present sample.

4.3 Comment:

“The bifactor model also deserves fuller interpretation. The results seem to indicate a strong general factor, yet the manuscript continues to discuss the three commitment dimensions as though their distinctiveness were unproblematic.”

Response:

Thank you for this important observation. We agree that the bifactor results indicate a strong general organizational commitment factor and that this required fuller interpretation.

The revised manuscript now clarifies that although affective commitment, normative commitment, and continuance commitment remain theoretically meaningful, their empirical distinctiveness in the present sample was more limited than a simple three-factor interpretation would suggest.

Specifically:

• the high omega hierarchical coefficient for the general factor (OmegaH = 0.836) suggested that the overall organizational commitment score behaved as largely unidimensional;

• whereas the relatively low omega hierarchical subscale coefficients indicated that some subdimensions retained limited reliable variance once the general factor was taken into account.

Continuance commitment retained comparatively more specific variance, although it remained psychometrically weaker than the general factor.

Accordingly, the revised manuscript now emphasizes that:

• the structural findings are interpreted primarily at the level of overall organizational commitment;

• while the three subdimensions are interpreted with greater caution.

The revised manuscript now explicitly states:

“While the three commitment dimensions remain theoretically meaningful, their empirical distinctiveness in the present sample was more limited than a simple three-factor interpretation would imply.”

These revisions were intended to strengthen construct validity interpretation and improve conceptual transparency.

5. Inferential Validity and Interpretation of Results

Comment:

“The interpretation of the findings sometimes goes beyond…and being more explicit about what the design allows the authors to claim and what it does not.”

Response:

We thank the reviewer for this important methodological observation. We agree that some of the original wording may have implied stronger directional or causal inference than our cross-sectional correlational design can support.

In response, we systematically revised the interpretive language throughout the manuscript.

Specifically:

• terms such as “predicts,” “influence,” “effect,” “driver,” “enhances,” “fosters,” and “shapes” were revised where necessary and replaced with associational language such as “is associated with,” “is related to,” “shows a significant association with,” and “accounts for variance in” where appropriate.

In addition:

• the Discussion and Conclusion sections now explicitly acknowledge the limitations associated with cross-sectional self-report data;

• and additional discussion regarding common method bias, perceptual alignment, and shared method variance was added.

The revised manuscript now states:

“As the findings are based on cross-sectional self-report data collected at a single point in time, the observed relationship may partially reflect shared perceptual or method-related variance.”

The Conclusion section also now explicitly states:

“Given the cross-sectional and correlational design of this study, the findings should not be interpreted as causal relationships.”

We also clarified that SEM was used to test theoretically specified structural associations among latent variables, but does not itself establish causality.

These revisions were intended to ensure that all claims remain proportionate to the methodological limitations of the study design.

6. Empirical Interpretation and Substantive Insight

Comment:

The manuscript reported findings statistically but did not sufficiently interpret their substantive meaning.

Response:

We substantially expanded the Discussion section to deepen the interpretation of the findings.

The revised manuscript now:

• explains why distributed leadership may be more strongly associated with organizational commitment than school type;

• interprets the findings through relational leadership, professional interaction, and social exchange processes;

• examines why continuance commitment demonstrated a comparatively weaker relationship;

• and integrates the UAE context more directly into the interpretation of the findings.

For example, the revised discussion now explains:

“This suggests that teachers’ lived professional experiences within schools may be shaped more strongly by everyday leadership interactions than by broader institutional distinctions between public and private schools.”

The manuscript also now discusses how distributed leadership may foster professional cohesion across culturally diverse school communities and how multicultural educational contexts may shape teachers’ perceptions of leadership and commitment.

These revisions were intended to move the discussion beyond statistical reporting toward stronger substantive interpretation.

7. Contextual Contribution (UAE Setting)

Comment:

The UAE context was described but not sufficiently integrated analytically into the findings.

Response:

We thank the reviewer for this valuable observation. The UAE context is now integrated much more directly throughout the manuscript.

Specifically:

• the Introduction and Problem Statement now more explicitly discuss workforce diversity and the coexistence of public and private educational systems;

• the Discussion sections now interpret findings in relation to multicultural teacher populations, contextual diversity, leadership practice, and institutional variation across emirates;

• and the Conclusion now explicitly positions the UAE as a culturally diverse educational setting in which distributed leadership may hold particular relevance.

The revised manuscript also now discusses:

• differences between Emirati, Arab, and non-Arab teachers;

• contextual variation across emirates;

• and how distributed leadership may support professional cohesion in culturally diverse schools.

These revisions were intended to strengthen the contextual contribution of the study and ensure that the UAE setting functions analytically rather than descriptively.

8. Methodological Transparen

Attachment Submitted filename: Response to Reviewers.docx

# Editor report: Decision Letter 1

**DOI:** 10.1371/journal.pone.0351540.r003

**Authors:** Yaser Mohammed Al-Worafi, Yaser Mohammed Al-Worafi

28 May 2026

Association between distributed leadership and teachers' organizational commitment: Evidence from public and private schools in the United Arab Emirates

PONE-D-26-09053R1

Dear Dr. Ibrahim,

We’re pleased to inform you that your manuscript has been judged scientifically suitable for publication and will be formally accepted for publication once it meets all outstanding technical requirements.

Within one week, you’ll receive an e-mail detailing the required amendments. When these have been addressed, you’ll receive a formal acceptance letter and your manuscript will be scheduled for publication.

An invoice will be generated when your article is formally accepted. Please note, if your institution has a publishing partnership with PLOS and your article meets the relevant criteria, all or part of your publication costs will be covered. Please make sure your user information is up-to-date by logging into Editorial Manager at Editorial Manager® and clicking the ‘Update My Information' link at the top of the page. For questions related to billing, please contact billing support.

If your institution or institutions have a press office, please notify them about your upcoming paper to help maximize its impact. If they’ll be preparing press materials, please inform our press team as soon as possible -- no later than 48 hours after receiving the formal acceptance. Your manuscript will remain under strict press embargo until 2 pm Eastern Time on the date of publication. For more information, please contact onepress@plos.org.

Kind regards,

Yaser Mohammed Al-Worafi

Academic Editor

PLOS One

Additional Editor Comments (optional):

Reviewers' comments:

# Editor report: Acceptance letter

**DOI:** 10.1371/journal.pone.0351540.r004

**Authors:** Yaser Mohammed Al-Worafi, Yaser Mohammed Al-Worafi

PONE-D-26-09053R1

PLOS One

Dear Dr. Ibrahim,

I'm pleased to inform you that your manuscript has been deemed suitable for publication in PLOS One. Congratulations! Your manuscript is now being handed over to our production team.

At this stage, our production department will prepare your paper for publication. This includes ensuring the following:

* All references, tables, and figures are properly cited

* All relevant supporting information is included in the manuscript submission,

* There are no issues that prevent the paper from being properly typeset

You will receive further instructions from the production team, including instructions on how to review your proof when it is ready. Please keep in mind that we are working through a large volume of accepted articles, so please give us a few days to review your paper and let you know the next and final steps.

Lastly, if your institution or institutions have a press office, please let them know about your upcoming paper now to help maximize its impact. If they'll be preparing press materials, please inform our press team within the next 48 hours. Your manuscript will remain under strict press embargo until 2 pm Eastern Time on the date of publication. For more information, please contact onepress@plos.org.

You will receive an invoice from PLOS for your publication fee after your manuscript has reached the completed accept phase. If you receive an email requesting payment before acceptance or for any other service, this may be a phishing scheme. Learn how to identify phishing emails and protect your accounts at https://explore.plos.org/phishing.

If we can help with anything else, please email us at customercare@plos.org.

Thank you for submitting your work to PLOS ONE and supporting open access.

Kind regards,

PLOS ONE Editorial Office Staff

on behalf of

Professor Yaser Mohammed Al-Worafi

Academic Editor

PLOS One
